# Does Physical Inactivity Aggravate Liver Ischemia–Reperfusion Injury? Evidence from a Preclinical Wistar Rat Model

**DOI:** 10.3390/biology15201850

**Published:** 2026-10-15

**Authors:** Miguel Anjos, Pedro Silva Oliveira, Diogo Duarte Carvalho, Ariane Flores, Sofhia Ramos, Rita Ferreira, Ana Isabel Padrão, Ana Reis-Mendes, Hélder Fonseca

**Affiliations:** 1 Research Centre in Physical Activity, Health and Leisure (CIAFEL), Faculty of Sport, University of Porto (FADEUP), 4200-450 Porto, Portugal; up201807240@fade.up.pt (P.S.O.); diogoac@fade.up.pt (D.D.C.); aflores@fade.up.pt (A.F.); anaisabelpadrao@gmail.com (A.I.P.); hfonseca@fade.up.pt (H.F.); 2 Laboratory for Integrative and Translational Research in Population Health (ITR), 4050-600 Porto, Portugal; 3 Nucleus of Research in Human Motricity Sciences, Universidad Adventista de Chile, Chillán 3780000, Chile; 4 School of Kinesiology and Health Science, The Muscle Health Research Centre, York University, Toronto, ON M3J 1P3, Canada; sramos14@northwell.edu; 5 Associated Laboratory for Green Chemistry (LAQV) of the Network of Chemistry and Technology (REQUIMTE), Department of Chemistry, University of Aveiro, 3810-193 Aveiro, Portugal; ritaferreira@ua.pt

**Keywords:** physically inactive, physically active, liver surgery, ischemia–reperfusion injury

## Abstract

Background: Ischemia–reperfusion injury (IRI) is a major cause of postoperative liver dysfunction after hepatic resection. Physical activity has been shown to protect against cardiac IRI but its effects on hepatic IRI are largely unknown. This study determined, in a preclinical rat model, whether physical activity modulates the hepatic response to ischemia–reperfusion. Methods: Twenty-eight male Wistar rats were assigned to four groups (*n* = 7) in a 2 × 2 design combining housing (physically inactive vs. voluntary running wheel) and surgery (30 min partial hepatic ischemia followed by 24 h reperfusion vs. sham). Liver injury, inflammation, apoptosis, oxidative stress, endothelial and mitochondrial markers were assessed. Results: Ischemia increased transaminases, ALP, bile acids and total bilirubin and reduced albumin, confirming IRI. The transaminase rise was similar in physically inactive and active animals (ALT, *p* = 0.867), indicating that physical activity did not prevent hepatocellular necrosis. However, physically active animals exhibited lower MPO activity, TNF-α, Bax/Bcl-2 ratio and caspase-8, together with higher citrate synthase activity, with significant surgical procedure × activity interactions for MPO (*p* = 0.035), TNF-α (*p* = 0.008) and Bax/Bcl-2 (*p* = 0.026). Oxidative stress, endothelial and mitochondrial markers, mitochondrial respiration and H_2_O_2_ emission were similar between groups. Conclusions: Physical activity did not reduce hepatocellular necrosis but attenuated inflammatory and apoptotic responses following hepatic IRI and increased liver mitochondrial content.

## Simple Summary

Liver surgery often requires temporary interruption of the hepatic blood supply, which can cause additional damage when blood flow is restored, a process known as ischemia–reperfusion injury. Although physical activity protects against ischemia–reperfusion injury in the heart, whether physical inactivity worsens the hepatic response to this injury remains unclear. To address this question, rats were housed for 25 weeks either in standard cages or with free access to a running wheel before undergoing partial hepatic ischemia followed by 24 h of reperfusion. Physical inactivity did not increase direct hepatocellular injury, as circulating markers of liver damage were comparable between physically inactive and active animals. However, physically inactive animals exhibited greater inflammatory and apoptotic responses following ischemia–reperfusion, including increased neutrophil-associated activity, inflammatory signaling, and cell-death markers. Conversely, physically active animals showed greater hepatic mitochondrial content, although mitochondrial function was not different between groups. These findings indicate that physical inactivity does not worsen the initial hepatocellular injury caused by hepatic ischemia–reperfusion but exacerbates the inflammatory and apoptotic responses to that injury. Thus, physical inactivity may represent a modifiable factor influencing the liver’s ability to respond to ischemia–reperfusion injury.

## 1. Introduction

Liver disease is responsible for over two million deaths yearly, representing approximately 4% of global deaths [1]. Many of these include cases of parasitic infection and primary or metastatic liver cancer, which are frequently managed through surgical resection [2]. Hepatic resection implies a period of ischemia, which is accomplished through the Pringle maneuver [3]. This allows safer liver resection without severe hemorrhage. However, longer ischemia time potentially increases liver damage [4]. Paradoxically, whilst reperfusion reinstates cellular respiration, the burst of upcoming O_2_ substantially increases oxidative stress due to the inability of dysfunctional mitochondria to adequately use O_2_ as a final electron acceptor in the respiratory chain, thereby exacerbating the damage initiated during ischemia [5]. Ischemia–reperfusion injury (IRI) is the main etiological factor underpinning postoperative liver damage and multiorgan dysfunction, significantly contributing to higher morbidity, mortality and length of hospital stay [6,7]. Mechanistically, IRI occurs thorough a plethora of mechanisms, including direct cellular damage, oxidative stress and severe liver inflammation [8]. IRI also triggers the release of pro-inflammatory cytokines [9] and damage-associated molecular patterns (DAMPS) that, through the circulation, lead to distant organ damage [9]. Associated endothelial dysfunction also impairs vasomotor regulation and promotes neutrophil adhesion and platelet aggregation, contributing to microthrombus formation and ineffective microcirculation [10]. The unfavorable prognosis of liver IRI is further worsened by the coexistence of comorbidities such as liver steatosis [11] or steatohepatitis [12], frequently present in these patients [13].

Beyond the local hepatic damage, hepatic IRI drives a systemic inflammatory cas-cade whose consequences extend well beyond the injured liver parenchyma, including remote-organ injury affecting the kidney and lung and, in severe cases, multiple-organ dysfunction [14,15]. In the context of partial hepatic resection, severe postoperative liver dysfunction may progress to post-hepatectomy liver failure, which is a major determinant of postoperative morbidity and mortality [16]. This complication has also been associated with increased 30- and 90-day postoperative mortality [17]. Consequently, the clinical sig-nificance of hepatic IRI is determined not only by the extent of local parenchymal injury but also by the magnitude of the subsequent inflammatory and cell-death responses, which may represent a therapeutically relevant window for intervention [15].

The mechanisms underlying hepatic IRI are not fully understood, and no effective prophylaxis currently exists [18]. In this context, exercise has been shown to protect against cardiac IRI [19]. Notoriously, a single exercise bout before an ischemic insult significantly reduces myocardial infarct size, which was attributable to enhanced antioxidant defenses, particularly superoxide dismutase (SOD) upregulation [20]. Short-term endurance training (3–5 days) also confers similar benefits [21] through stimulation of mitochondrial adaptations and antioxidant activity [19]. Additionally, increased expression of mitochondrial ATP-sensitive potassium channels and improved endothelial function may also mechanistically contribute to exercise-induced IRI protection [22,23]. Nevertheless, patients with liver disease experience significant fatigue and are often advised to rest [24]. In many cases, they are not actively encouraged to engage in physical activity, resulting in a physically inactive lifestyle prior to surgery which, considering the evidence regarding the potential benefits of physical activity in this context, could potentially worsen IRI complications [24].

Given the aforementioned available evidence, the central hypothesis of this study is that physical inactivity may exacerbate the severity of hepatic IRI. If this hypothesis holds true, considering the high number of liver surgeries performed annually [25], implementation of preoperative exercise programs could yield significant benefits for patient outcomes and healthcare systems. Therefore, this study aims to investigate the impact of physical inactivity on markers of liver IRI severity in preclinical rat models.

## 2. Materials and Methods

### 2.1. Animals and Experimental Groups

For this study, the experimental model was implemented mimicking two paradigms of physical activity. A model of physically active animals was implemented by housing rats in cages with access to a running wheel (Tecniplast, Buguggiate, Italy), where they could freely express their typically active behavior. In opposition, physically inactive animals were housed in conventional laboratory cages without access to a running wheel, their activity being restricted to the limited cage floor area (floor area 800 cm^2^).

**Figure 1:**
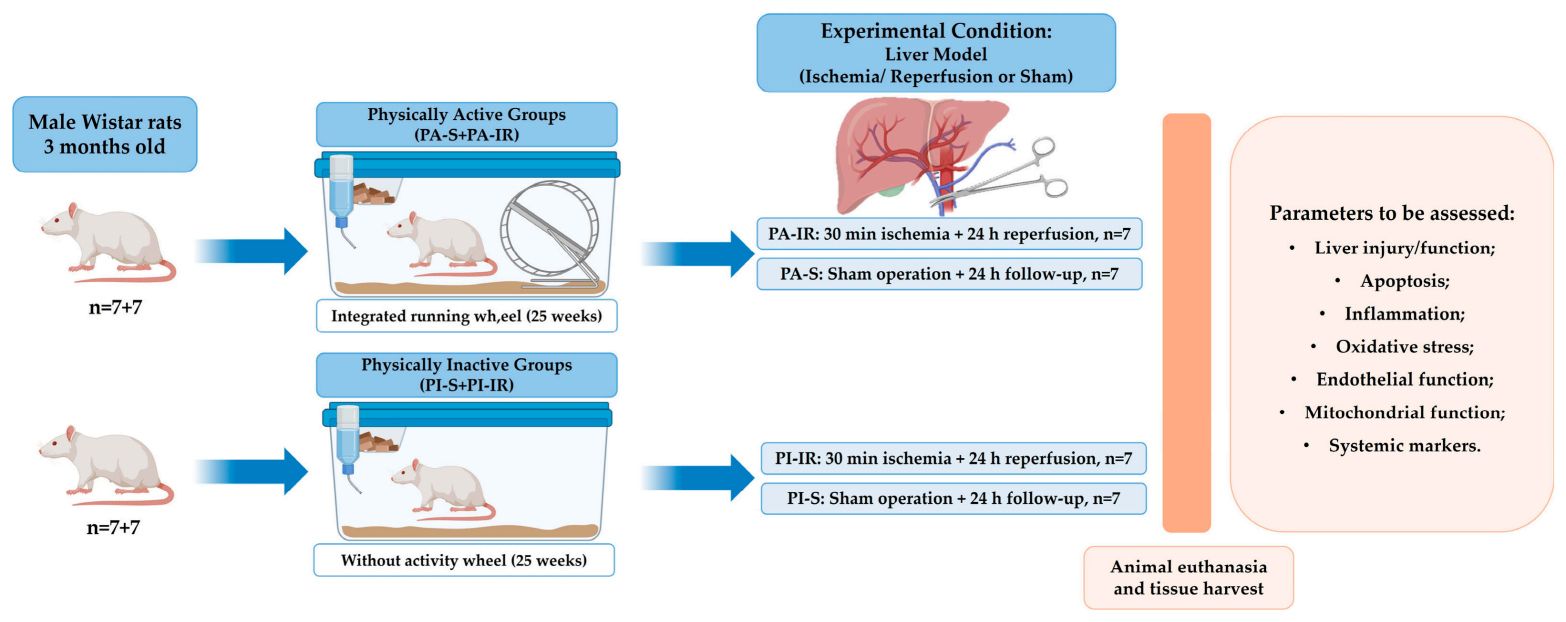
Overview of experimental design. Male Wistar rats (3 months old; *n* = 7 per group) were housed for 25 weeks with a running wheel (physically active, PA-S+PA-IR) or in a standard physical inactivity cage (physically inactive, PI-S+PI-IR), then they underwent 30 min hepatic ischemia + 24 h reperfusion (*n* = 7) or sham + 24 h follow-up (*n* = 7). Created in BioRender. Filipa Mendes, A. (2026) https://BioRender.com/p2c70lt.

Twenty-eight male Wistar rats aged 12 weeks (Charles River Laboratories, L’Arbresle, France) were housed under controlled environmental conditions (12:12 h inverted light-dark cycle, 24 ± 2 °C, 50 ± 10% humidity) with access to a standard laboratory diet (4RF21^®^, Mucedola, S.r.l., Settimo Milanese, Italy) and water ad libitum. After a 2-week quarantine period, animals were weighed and stratified into four weight-based blocks of seven animals, after which they were randomly allocated within each block to one of four experimental groups (*n* = 7/group): physically inactive—sham (PI-S), physically inactive ischemia–reperfusion (PI-IR), physically active—sham (PA-S), and physically active ischemia–reperfusion (PA-IR). This procedure ensured a balanced distribution of baseline body weight across experimental groups. The assigned housing conditions were maintained for 25 weeks, during which running wheel activity was continuously recorded in the physically active groups (Figure 1). Wheel activity was recorded automatically and continuously (24 h/day) throughout the entire 25-week intervention by the digital counter integrated in the Tecniplast running wheel system, which registers each wheel revolution in real time; from the revolution counts, daily distance (m/day) was derived for each animal (see Section 3.1). Sample size was determined based on the effect size identified in a previous study evaluating liver damage score between exercised and sedentary animals. Calculations were performed assuming α = 0.05 and a statistical power of 80%, and the minimum number of animals required per group was estimated accordingly. Food intake was recorded weekly and body weight every two weeks during the study, with all animals housed individually. All procedures were performed in accordance with the guidelines for the Care and Use of Laboratory Animals in Research recommended by the European Federation of Associations for the Science of Laboratory Animals (FELASA). The Local Ethics Committee approved the study protocol (CEFADE 19-2023), according to the EU directive (2010/63/EU) and Portuguese law (DL 113/2013). Humane endpoints were established *a priori*. Animals showing major unexpected adverse effects, including severe discomfort, infection, hemorrhage or wound dehiscence, would be immediately euthanized. No adverse events requiring euthanasia or exclusion occurred during the study.

### 2.2. Implementation of Liver IR Models

**Figure 2:**
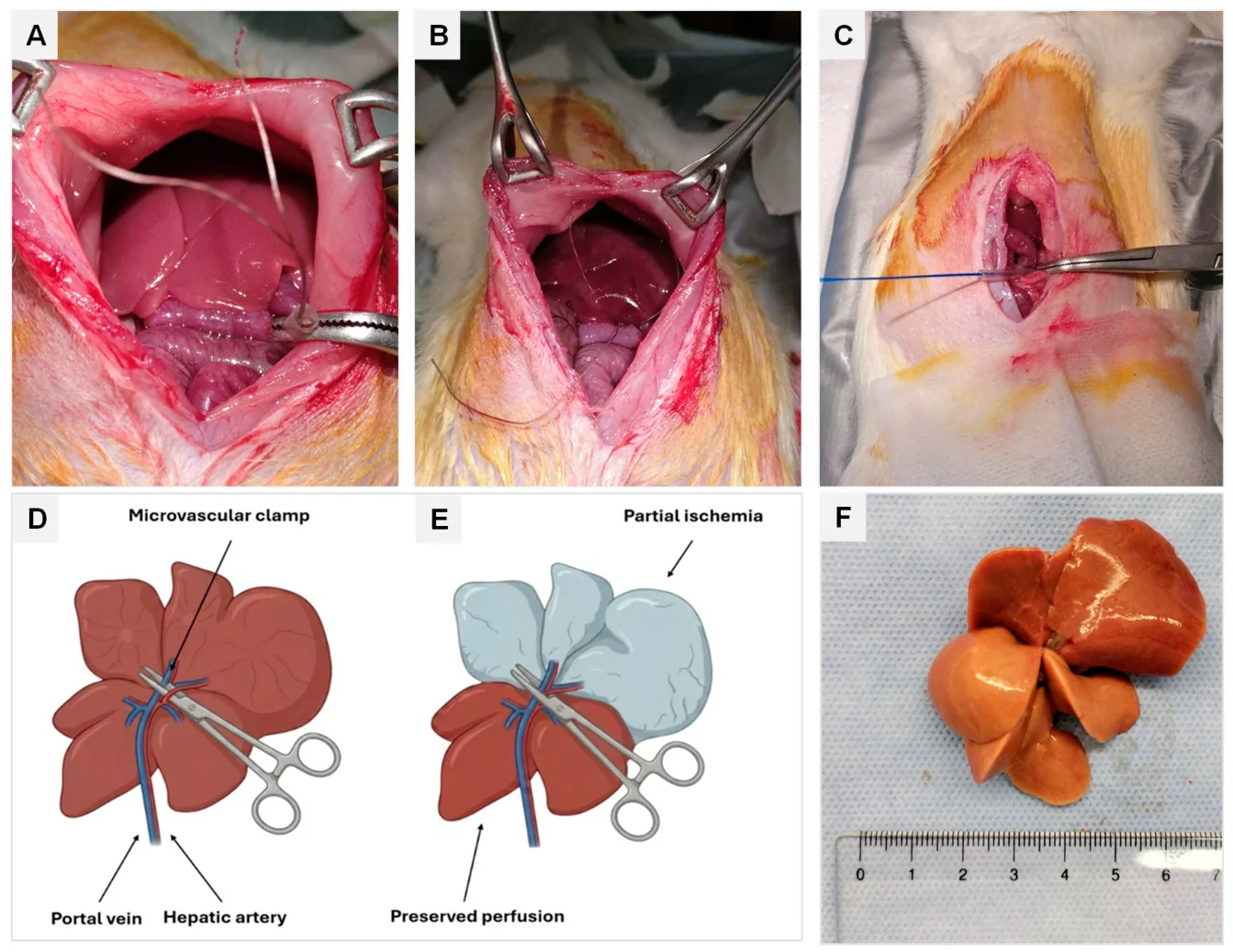
Surgical procedure used to induce partial hepatic ischemia–reperfusion in the Wistar rat model, and macroscopic confirmation of effective ischemia. (**A**) Intra-operative view after midline laparotomy showing the exposed liver prior to vascular clamping, with hepatic lobes exhibiting a homogeneous physiological red coloration. (**B**) Intra-operative view during partial hepatic ischemia, showing the characteristic dark violaceous discoloration of the clamped median and left lobes reflecting effective portal-pedicle occlusion. (**C**) Closure of the abdominal wall by continuous suture at the end of the surgical procedure. (**D**) Schematic representation of the microvascular clamp applied to the portal pedicle, comprising the hepatic artery (red) and portal vein (blue). (**E**) Schematic representation of the resulting partial-ischemia pattern, in which the clamped lobes become pale (median and left) while the right and caudate lobes retain their perfusion. (**F**) Representative ex vivo photograph documenting the macroscopic demarcation between the pale ischemic lobes (median and left) and the darker, non-ischemic lobes (right and caudate) with preserved perfusion, consistent with the model illustrated in (**E**). Panels (**D**,**E**) were created in BioRender. Filipa Mendes, A. (2026) https://BioRender.com/d4gijrg.

At the end of the 25-week period of intervention, PA-IR and PI-IR groups underwent a 30 min partial liver ischemia followed by 24 h of reperfusion while PA-S and PI-S groups underwent a sham operation. Briefly, animals were anesthetized with sevoflurane (6–7% induction, 2–3% maintenance) in 2 L/min O_2_. Animals were then placed supine over a heating pad (37 °C), limbs secured, and a ventral trichotomy was performed. After wiping the abdominal skin with antiseptic solution, a median xiphopubic laparotomy was performed. The portal vein ramus to the caudate and right hepatic lobes were identified and a 2.0 temporary nylon suture was placed around the hepatic vein and artery to induce ischemia. This was confirmed by the immediate paling of the median and left hepatic lobes (Figure 1). Ischemia was maintained for 30 min, at the end of which the suture was removed. Reperfusion was confirmed by the immediate appearance of a darker color in the median and left hepatic lobes. The abdominal incision was closed and the animal placed in a warm recovery cage. Tramadol (12.5 mg/kg, IP; Tramal, Grünenthal, Aachen, Germany) was administered for analgesia [26]. PI-S and PA-S groups underwent the same procedure without tightening the hepatic vessel sutures, thereby maintaining liver blood flow. Effective induction of IRI was confirmed macroscopically during surgery (immediate paling of the median and left lobes upon clamping and return of a darker coloration upon suture release) (Figure 2) and further supported histologically on hematoxylin and eosin (H&E)-stained liver sections (see Section 3.2).

### 2.3. Euthanasia and Necropsy Procedures

Twenty-four hours after reperfusion or the sham procedure, animals were sacrificed with an IP overdose of ketamine (Nimatek 100 mg/mL, Dechra, Northwich, UK) and xylazine (Rompun 2%,Bayer, Leverkusen, Germany) for blood and liver sample collection. All procedures were performed between 08:00 and 14:00 to minimize circadian-related variability. Five mL of venous blood was collected from the abdominal vena cava into heparin-coated tubes and immediately centrifuged (10,000× *g*, 10 min, 4 °C) and the plasma was separated, aliquoted and stored at −80 °C. The liver was harvested, cleared of blood and surrounding tissue and weighed. Samples from the median and left lobes were collected and either stored at −80 °C or immediately prepared for analysis according to each specific protocol.

### 2.4. Histological Analysis

For histological assessment, six liver fragments of approximately 5 mm^3^ were obtained per animal, three from the median lobe and three from the left lobe, sampled from anatomically comparable regions across animals to ensure consistency. Fragments were immediately immersion-fixed in 4% (*w*/*v*) buffered formaldehyde (ref. 252931.1315, PanReac AppliChem, Barcelona, Spain) for 48 h. Fixed tissue was subsequently dehydrated through a graded ethanol series (ref. E/0650DF/C17, Fisher Chemical, Loughborough, UK), cleared in xylene (ref. 6615, Thermo Scientific, Waltham, MA, USA) and paraffin-embedded (ref. 8336, Thermo Scientific, Waltham, MA, USA). Five µm sections were cut on a rotary microtome (Leica RM2125 RT; Leica Microsystems, Nussloch, Germany), mounted on silane-coated glass slides, deparaffinized in xylene, rehydrated through reverse ethanol steps, and stained with hematoxylin (ref. HHS32, Sigma-Aldrich, St. Louis, MO, USA) and eosin (ref. HT110132, Sigma-Aldrich, St. Louis, MO, USA). Slides were coverslipped using DPX mounting medium (ref. 06522, Sigma-Aldrich, St. Louis, MO, USA). The slides were analyzed in a Carl Zeiss Imager A1 light microscope and images were recorded with a coupled AxioCam MRc 5 digital camera (Oberkochen, Germany). Representative images were obtained from selected fields showing histological features of interest, including focal hepatocyte coagulative necrosis and sinusoidal congestion, at 10× and 40× magnification for all groups, with additional 100× magnification used for the ischemia–reperfusion groups. For semi-quantitative assessment, all H&E sections were evaluated by an observer blinded to group allocation in 10 non-overlapping fields per animal at 40× magnification, covering both the median and left lobes. Hepatic injury was graded according to the Suzuki score [27], in which sinusoidal congestion, hepatocyte cytoplasmic vacuolization and parenchymal necrosis are each scored from 0 (absent) to 4 (severe), yielding a total score of 0–12. Each parameter was graded in every field, and the median of the 10 fields was taken as the value of each animal. In addition, each lesion (necrosis, sinusoidal congestion, cytoplasmic vacuolization and inflammatory infiltrate) was classified as present or absent in each animal, a lesion being considered present when graded ≥ 1 or, for inflammatory infiltrate, when observed in at least one field.

### 2.5. High-Resolution Respirometry

Approximately 8 mg of liver tissue was collected immediately after animal euthanasia and mechanically homogenized using the PBI-Shredder O2k (Oroboros Instruments, Innsbruck, Austria) [28]. Samples were then transferred to the O2k respirometry chamber containing 2 mL of mitochondrial respiration medium MiR05 (ID: 60101-01, Lot: 20J01923; Oroboros Instruments), pre-equilibrated at 37 °C. Fluorometry was concomitantly performed during oxygen consumption (O_2_ flux) for hydrogen peroxide (H_2_O_2_) production detection, according to the SUIT-009 AmR mt D021 protocol (Table S1), specifically adapted for tissue homogenates.

Prior to substrate and inhibitor titration, the chamber was supplemented with diethylenetriaminepentaacetic acid (DTPA, to chelate transition metals and reduce nonspecific fluorescence background), horseradish peroxidase (HRP), SOD, and Amplex UltraRed (AmR) to enable detection of H_2_O_2_ through the formation of the fluorescent product resorufin. The titration protocol began with the addition of succinate to support reverse electron transfer (RET)-associated H_2_O_2_ production in the LEAK state (absence of adenylates), followed by ADP to stimulate oxidative phosphorylation (OXPHOS). Pyruvate was then added to activate the NADH-linked pathway and allow convergent electron flow through complexes I and II (NS-pathway) under OXPHOS conditions. Subsequently, rotenone was used to inhibit complex I, isolating complex II-supported respiration, and antimycin A was added to inhibit complex III and determine residual oxygen consumption (ROX), which was subtracted from raw values to correct mitochondrial O_2_ flux. Multiple H_2_O_2_ calibrations were performed throughout the experiment to account for sensitivity drift of the Amplex UltraRed assay over time and following reagent additions. Oxygen and H_2_O_2_ fluxes were normalized to the mass of tissue used and corrected for non-mitochondrial background activity. All measurements were conducted under continuous stirring at 750 rpm and maintained at 37 °C.

### 2.6. Biochemical Analysis

#### 2.6.1. Tissue Homogenization and Total Protein Quantification

Liver tissue samples were homogenized on ice using a glass Potter-Elvehjem homogenizer equipped with a Teflon pestle in ice-cold RIPA buffer (EMD Millipore, Cat. No. 20-188), supplemented with protease inhibitors (Sigma-Aldrich, P8340) and phosphatase inhibitors (Sigma-Aldrich, P0044 and P5726). Protein concentration was quantified using the DC Protein Assay Kit (Cat. No. 5000111, Bio-Rad^®^, Hercules, CA, USA), following the manufacturer’s instructions, with bovine serum albumin (BSA) as the protein standard. Absorbance was recorded at 750 nm using a Multiskan GO microplate spectrophotometer (Thermo Fisher Scientific^®^, Northumberland, UK).

#### 2.6.2. Western Blot Analysis

Twenty µg of each sample was separated by electrophoresis on 10% or 12.5% SDS-PAGE gels prepared according to Laemmli [29] and transferred onto nitrocellulose membranes (Whatman^®^, Protran^®^, Merck, Marlborough, MA, USA) using the Trans-Blot^®^ Turbo system (Bio-Rad, Hercules, CA, USA) at 2500 mA and 25 V for 7 min (Mixed MW protocol). After transfer, membranes were stained with Ponceau S and digitized as a total protein loading control [30] and were then cut horizontally according to the molecular weight of each target, so that several proteins could be detected on the same membrane. Membranes were blocked for 10 min with EveryBlot Blocking Buffer (#12010020, Bio-Rad, Hercules, CA, USA) and incubated overnight at 4 °C with the appropriate primary antibodies diluted in EveryBlot Blocking Buffer: rabbit polyclonal anti-mtTFA (sc-376672; 1:200), mouse monoclonal anti-MFN2 (sc-515647; 1:1000), mouse monoclonal anti-MFN1 (sc-166644; 1:750), rabbit polyclonal anti-PGC1-α (ab54481; 1:1000), mouse monoclonal anti-Bcl-2 (ab16904; 1:1000), rabbit polyclonal anti-Bax (ab32503; 1:1000), rabbit polyclonal anti-Calpain-2 (ab39165; 1:1000), rabbit polyclonal anti-Caspase-8 (ab25901; 1:1000), rabbit polyclonal anti-Caspase-3 (ab13847; 1:2000), rabbit polyclonal anti-MPO (ab208670; 1:2000), rabbit polyclonal anti-TNF-α (ab6671; 1:500), mouse monoclonal anti-NF-κB p50 (sc-114; 1:750), rabbit polyclonal anti-Glutathione peroxidase 1 (ab22604; 1:2000), rabbit polyclonal anti-SOD2 (ab13534; 1:1000), rabbit monoclonal anti-VCAM1 (ab134047; 1:2000), rabbit monoclonal anti-eNOS (ab199956; 1:1000), rabbit polyclonal anti-CD68 (ab125212; 1:1000), rabbit monoclonal anti-CD163 (ab182422; 1:1000), rabbit polyclonal anti-Catalase (ab16731; 1:2000), mouse monoclonal anti-Endothelin 1 (ab2786; 1:500), mouse monoclonal anti-ICAM1 (ab171123; 1:250) and rabbit multiclonal anti-iNOS (ab283655; 1:1000). Following washes, membranes were incubated with HRP-conjugated anti-mouse (ab205719) or anti-rabbit (ab6721) secondary antibodies diluted 1:5000 in EveryBlot Blocking Buffer. Bands were detected using Clarity Western ECL Substrate (Bio-Rad) and digitized with the ChemiDoc Imaging System (version 2.3.0.07, Bio-Rad). Band intensities were quantified using Image Lab (version 6.1, Bio-Rad). The intensity of each band was normalized to the Ponceau S signal of the same lane, as recommended [31]; targets detected on the same membrane were therefore normalized to the same Ponceau S image. All Western blot targets reported here were quantified in liver tissue homogenates, and the corresponding membrane images and Ponceau S normalization are provided in Supplementary Figure S1. Total protein normalization by Ponceau S was used instead of a single housekeeping protein because the expression of housekeeping proteins may vary under experimental conditions such as IR and physical activity [32,33,34,35].

#### 2.6.3. Slot-Blot Analysis

3-Nitrotyrosine and 4-Hydroxynonenal Forty µg of total protein per sample was diluted in Tris-buffered saline (TBS). Subsequently, 100 µL of each sample was applied to nitrocellulose membranes (Amersham™ Protran™, 0.45 µm, GE Healthcare, Chicago, IL, USA) using a slot-blot vacuum system (Bio-Rad Laboratories, Hercules, CA, USA; Cat. No. 1706542). Membranes were previously activated by immersion in 10% methanol for 5 s and equilibrated in distilled water prior to sample application. Following protein immobilization, membranes were blocked for 1 h in 5% (*w*/*v*) non-fat dry milk in Tris-buffered saline with tween-20 (TBS-T) (100 mM Tris, 1.5 mM NaCl, pH 8.0, 0.5% Tween-20). Membranes were then incubated for 1 h at room temperature with primary antibodies diluted in the same blocking solution: mouse monoclonal anti-3-nitrotyrosine (3-NT) (MAB5404; 1:1000) or rabbit polyclonal anti-4-hydroxynonenal (4-HNE) (ab46545; 1:2000). After three washes of 10 min each in TBS-T, membranes were incubated with HRP-conjugated secondary antibodies (anti-mouse IgG or anti-rabbit IgG, 1:5000) in blocking solution for 45 min at room temperature with shaking. Signal detection was performed using Clarity™ Western ECL Substrate (Bio-Rad). Images were captured with the ChemiDoc Imaging System (version 2.3.0.07, Bio-Rad, Hercules, CA, USA) and band intensities quantified using Image Lab (version 6.1, Bio-Rad, Hercules, CA, USA).

#### 2.6.4. Carbonylated Proteins

Protein carbonylation was assessed using a modified version of the method described by Robinson et al. [36]. Briefly, homogenate samples containing 40 µg of protein were mixed with an equal volume of 12% (*w*/*v*) SDS and 2 volumes of 20 mM 2,4-dinitrophenylhydrazine (DNPH) prepared in 10% (*v*/*v*) trifluoroacetic acid. After 30 min of incubation in the dark at room temperature, the reaction was stopped by adding 1.5 volumes of 2 M Tris containing 18% (*v*/*v*) β-mercaptoethanol. Dilution (1:200 in TBS) was prepared, and 100 µL of each was loaded onto nitrocellulose membranes (Amersham™ Protran™, 0.45 μm, GE Healthcare) under vacuum using a slot-blot apparatus. Prior to sample loading, membranes were activated in 10% (*v*/*v*) methanol for 5 s and equilibrated in distilled water. Membranes were blocked for 1 h in 5% (*w/v*) non-fat dry milk in TBS-T (100 mM Tris, 1.5 mM NaCl, pH 8.0, 0.5% Tween-20) and then incubated with the primary antibody (rabbit anti-DNP, 1:1000; MAB2223, Millipore, Burlington, MA, USA) diluted in blocking solution for 45 min at room temperature with shaking. After washing (3 × 10 min) with TBS-T, membranes were incubated for 30 min with HRP-conjugated secondary antibody (anti-rabbit IgG, 1:5000 in blocking solution). Immunoreactive spots were visualized using Clarity™ Western ECL Substrate (Bio-Rad), and digital images were acquired using the ChemiDoc Imaging System (version 2.3.0.07, Bio-Rad, Hercules, CA, USA). Densitometric analysis was performed with Image Lab (version 6.1, Bio-Rad, Hercules, CA, USA).

#### 2.6.5. Citrate Synthase (CS) Activity

CS activity was measured in sample homogenates following Coore et al. [37]. Samples were homogenized in a mild lysis buffer (50 mM Tris-HCl, pH 7.4, containing 0.1% Triton X-100 and protease inhibitors) to preserve enzymatic activity, avoiding ionic detergents that could denature the enzyme. CoASH released from the reaction of acetyl-CoA (Sigma-Aldrich: A2056) with oxaloacetate (Sigma-Aldrich: O7753) was measured by its reaction with 5,5’-dithiobis-(2-nitrobenzoic acid) (DTNB, Sigma-Aldrich: D8130) at 412 nm (ε = 13.6 mM^−1^cm^−1^). Total CS activity was expressed in nmol·min^−1^·mg^−1^ protein on each sample.

### 2.7. Plasma Chemical Analysis

#### 2.7.1. Myeloperoxidase (MPO) Activity

MPO activity was measured in plasma samples using a colorimetric MPO Assay Kit (MAK516, Sigma-Aldrich) based on the oxidation of chloride ions in the presence of hydrogen peroxide. In this method, MPO catalyzes the formation of hypochlorous acid, which reacts with taurine to produce taurine chloramine. This intermediate then reacts with the chromophore 2-nitro-5-thiobenzoic acid (TNB), leading to the formation of the product DTNB, resulting in a decrease in absorbance at 412 nm. The decrease in absorbance was monitored over time, and the reaction rate was calculated from the linear portion of the curve. MPO activity was expressed as nmol·min^−1^·mL^−1^ of plasma, where one unit corresponds to the amount of enzyme that consumes 1 nmol of TNB per minute at 25 °C.

#### 2.7.2. Plasma Analysis

Plasma samples were promptly processed to assess liver function and related biochemical parameters using the fully automated clinical chemistry analyzer Accent S 120 (PZ Cormay S.A., Łomianki, Poland). All biomarkers were quantified spectrophotometrically according to the manufacturer’s validated protocols. The monitored absorbance was directly proportional to the catalytic activity of the enzymes, expressed in U/L (AST, ALP, CK and amylase), or to the concentration of the remaining analytes (albumin, g/L; creatinine, glucose and lactate, mg/dL; bile acids, µmol/L). The following dedicated ACCENT-200 reagent kits were used: aspartate aminotransferase (AST; ACCENT-200 ASAT, REF 7-214), alkaline phosphatase (ALP; ACCENT-200 ALP, REF 7-212), alanine aminotransferase (ALT; ACCENT-200 ALAT, REF 7-216), albumin (ACCENT-200 ALBUMIN, REF 7-238), amylase (ACCENT-200 AMILASE, REF 7-276), direct bilirubin (ACCENT-200 BIL DIRECT, REF 7-248), total bilirubin (ACCENT-200 BIL TOTAL, REF 7-245), creatine kinase (CK; ACCENT-200 CK, REF 7-220), creatinine (ACCENT-200 CREATININE, REF 7-233), glucose (ACCENT-200 GLUCOSE, REF 7-201), lactate (ACCENT-200 LACTATE, REF 7-266), and bile acids (ACCENT-200 BILE ACIDS, REF 7-198). For all analytes except bile acids, quantification was based on a multi-point calibration curve constructed with the CORMAY Multicalibrator Level 1 (REF 5-176) and Level 2 (REF 5-177), and analytical accuracy and precision were verified by internal quality control using CORMAY Serum HN (REF 5-172) and CORMAY Serum HP (REF 5-173) control materials. Bile acids were calibrated separately with the CORMAY Bile Acids Calibrator (REF 3-125) and controlled with the CORMAY Bile Acids Controls (REF 5-149). All procedures were performed in accordance with the manufacturer’s recommendations.

### 2.8. Statistical Analysis

Statistical analyses were performed and figures generated using GraphPad Prism version 11 (GraphPad Software, San Diego, CA, USA). The significance level was set at *p* < 0.05 (two-tailed), and data are presented as mean ± standard deviation.

Descriptive statistics were computed for all relevant variables. Prior to inferential testing, outliers were screened using the ROUT method (Q = 1%) and normality was assessed with the Shapiro–Wilk test. Variables that violated the assumption of normality were subjected to a natural logarithmic transformation (x + 1, to accommodate values close to zero); those that still failed to meet parametric assumptions were analyzed non-parametrically, as specified below.

For variables meeting parametric assumptions, a two-way ANOVA examined the main effects of physical activity condition (inactive vs. active) and surgical procedure (sham vs. ischemia) and their interaction.

The study followed a 2 × 2 factorial design, and Table 1 reports the two main effects derived from it. The main effect of surgical procedure (PI-IR+PA-IR vs. PI-S+PA-S) tests whether the IR insult itself altered each parameter, irrespective of activity condition. The main effect of physical activity condition (PA-S+PA-IR vs. PI-S+PI-IR) tests whether habitual physical activity altered each parameter, irrespective of surgical procedure. The central question of the study, namely whether physical inactivity modifies the hepatic response to IR, corresponds to the physical activity × surgical procedure interaction, which was therefore formally tested in each two-way ANOVA model. Main effects were interpreted as independent contributions of each factor only when the interaction was not significant. When the interaction reached significance, main effects are reported for completeness, but the effect of physical activity was interpreted within each surgical condition, based on the pairwise comparisons among the four experimental groups (PI-IR vs. PA-IR and PI-S vs. PA-S). To further characterize differences among the four experimental groups (PI-S, PA-S, PI-IR, PA-IR), a one-way ANOVA followed by Tukey’s *post hoc* test was additionally performed. The between-group comparisons displayed in the figures are derived from this analysis. Effect sizes for ANOVA models were estimated using partial eta-squared (η^2^_p_), interpreted as small (0.05–0.25), moderate (0.25–0.64), or strong (>0.64) [38]. For variables that did not meet parametric assumptions after transformation, the main effects of physical activity condition and surgical procedure were assessed individually using the Mann–Whitney U test, and the four experimental groups were compared using the Kruskal–Wallis test followed by Dunn’s *post hoc* test. The specific test applied to each variable is indicated in the corresponding figure legend. Outliers were removed only when attributable to technical constraints during laboratory analysis, and all such cases are explicitly reported for each affected variable.

Suzuki scores were compared among the four groups using the Kruskal–Wallis test followed by Dunn’s *post hoc* test. The incidence of each histological lesion was compared using a two-sided Fisher’s exact test between sham and IR animals within each activity condition (PI-S vs. PI-IR; PA-S vs. PA-IR), between PI-IR and PA-IR, and between pooled sham and pooled IR animals; Fisher’s exact test *p*-values are reported without adjustment for multiple comparisons.

Body mass, food intake, and distance covered in the running wheel were compared across the 25-week period by two-way ANOVA followed by Šídák’s multiple comparisons test.

## 3. Results

### 3.1. Body Mass, Food Intake and Voluntary Physical Activity

**Figure 3:**
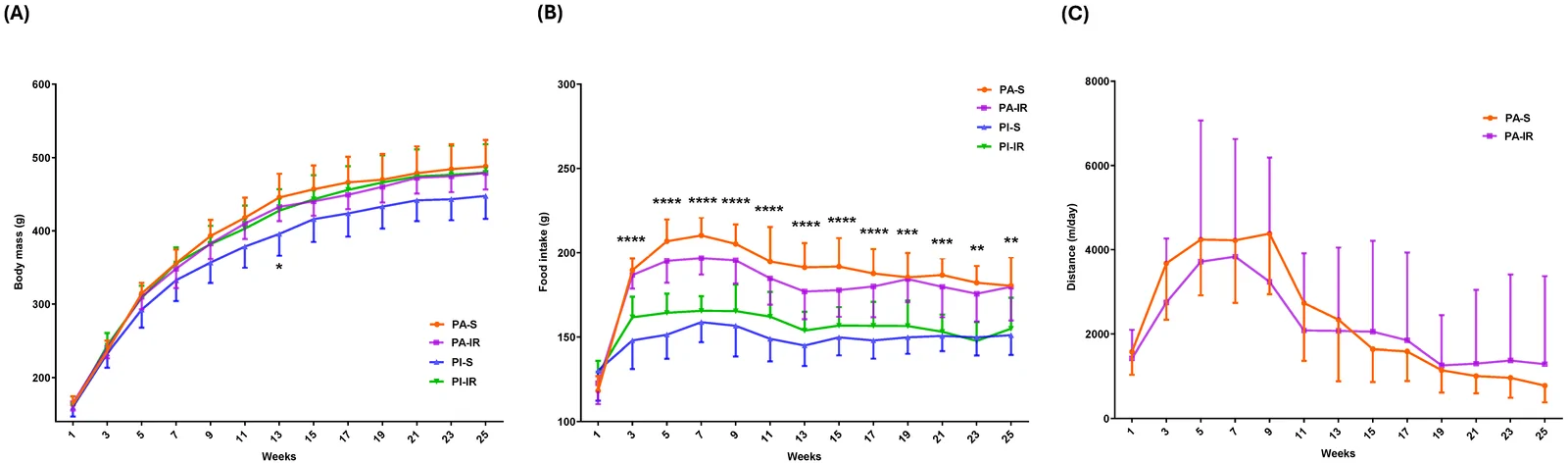
Body mass, food intake, and locomotor activity over 25 weeks in the physically active—sham (PA-S), physically active ischemia–reperfusion (PA-IR), physically inactive—sham (PI-S), and physically inactive ischemia–reperfusion (PI-IR) groups. Data are presented as mean ± standard deviation (*n* = 7/group), analyzed by two-way ANOVA followed by Šídák’s multiple comparisons post-test. (**A**) Body mass (g). The asterisk indicates a significant difference between PA-S vs. PI-S (* *p* < 0.05). (**B**) Food intake (g). Asterisks indicate significant differences between PA-S vs. PI-S (** *p* < 0.01; *** *p* < 0.001; **** *p* < 0.0001); the other significant comparisons (PA-S vs. PI-IR, PA-IR vs. PI-S, and PA-IR vs. PI-IR) followed a similar pattern and are detailed in Table S2. (**C**) Distance traveled (meters per day) for the PA-S and PA-IR groups with no significant differences observed between groups at any time point. Individual values for each animal are provided in Table S3.

At baseline there were no weight differences between groups. During the experiment, body mass increased similarly in all groups (Figure 3A). Average weekly food intake was higher in the physically active groups (PA-S, PA-IR) compared to the physically inactive groups (PI-S, PI-IR) (Figure 3B). Running distance in the activity wheel was similar between the two physically active groups (PA-S, PA-IR) throughout the pre-surgical intervention period (Figure 3C, individual data for each animal are provided in Table S3).

### 3.2. Histology Analysis of Liver Tissue

**Figure 4:**
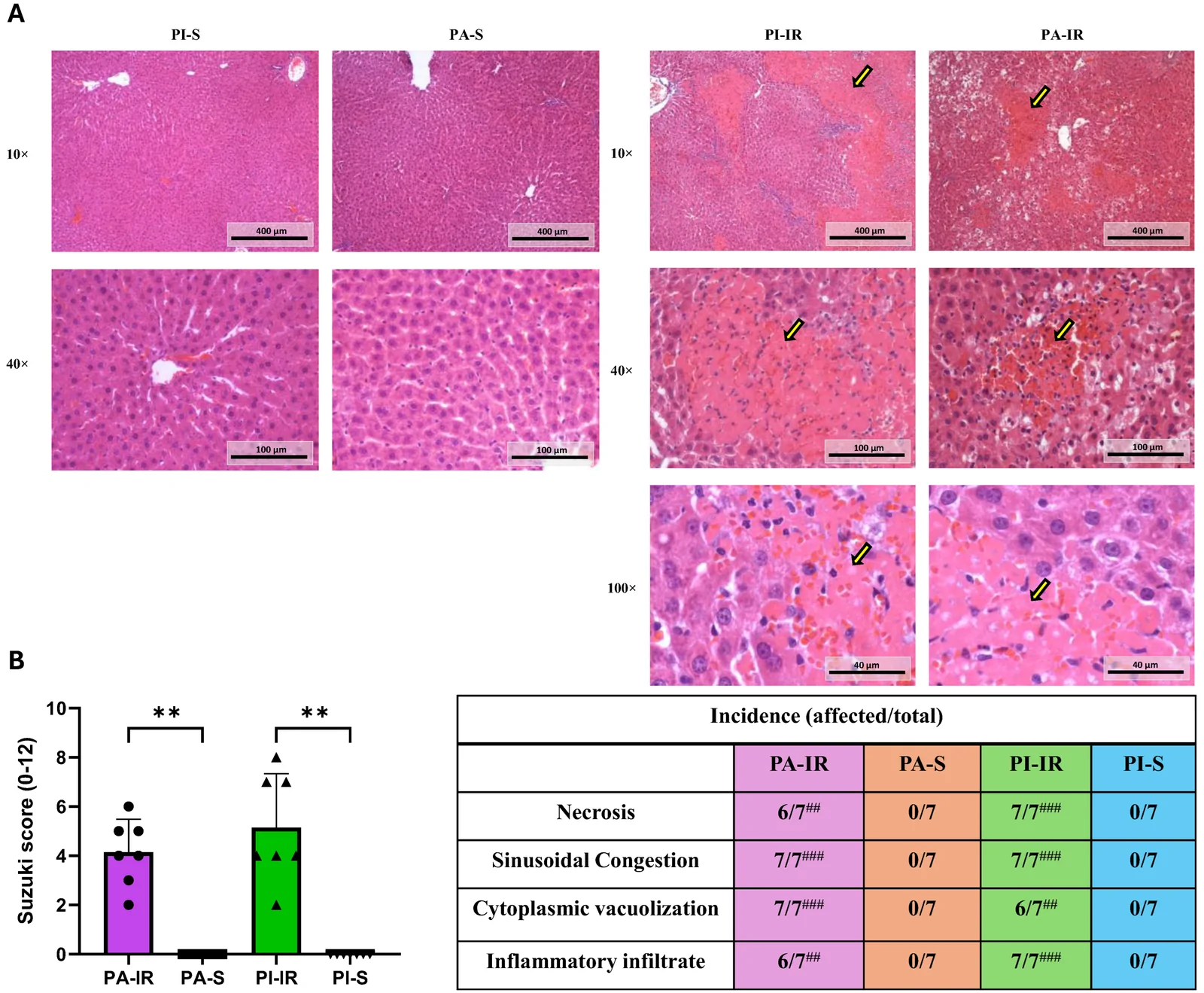
Hepatic histopathology microphotographs of Wistar rats after hematoxylin and eosin (H&E) staining, from the four experimental groups: PI-S (physically inactive—sham), PA-S (physically active—sham), PI-IR (physically inactive ischemia–reperfusion), and PA-IR (physically active ischemia–reperfusion). (**A**) Representative light micrographs from sham-operated animals (PI-S, PA-S) presented preserved lobular architecture with hepatocytes of normal morphology, while the ischemia–reperfusion animals (PI-IR, PA-IR) displayed focal hepatocyte coagulative necrosis and sinusoidal congestion (yellow arrows). Images were taken at 10× and 40× magnification for all groups; for ischemia–reperfusion groups, additional images were taken at 100× magnification. Scale bars = 400 μm (10×), 100 μm (40×), and 40 μm (100×). Representative images of 3 animals per group. (**B**) Suzuki score (sinusoidal congestion, cytoplasmic vacuolization and necrosis; total score 0–12) of each animal; bars represent median and interquartile range (*n* = 7 per group); Kruskal–Wallis test followed by Dunn’s *post hoc* test (** *p* < 0.01). The incidence of each lesion (affected/total animals) is shown on the right and was compared with the respective sham group by two-sided Fisher’s exact test (^##^ *p* < 0.01; ^###^ *p* < 0.001).

Histological examination of H&E-stained liver sections confirmed the successful in-duction of the ischemia–reperfusion model (Figure 4). Sham-operated animals (PI-S and PA-S) displayed preserved lobular architecture with hepatocytes of normal morphology and no evidence of necrosis, sinusoidal congestion, cytoplasmatic vacuolization or inflammatory infiltrate. In contrast, ischemia–reperfusion animals (PI-IR and PA-IR) presented focal hepatocyte coagulative necrosis and sinusoidal congestion, accompanied by cytoplasmic vacuolization and inflammatory infiltrate, predominantly in the median and left hepatic lobes subjected to vascular occlusion.

The Suzuki score was higher in IR than in sham animals [median: PI-S, 0 (0–0); PA-S, 0 (0–0); PI-IR, 4 (4–7); PA-IR, 4 (3–5); *p* < 0.001, Kruskal–Wallis test; PI-S vs. PI-IR, *p* = 0.002; PA-S vs. PA-IR, *p* = 0.008; Dunn’s test], with no significant difference between PI-IR and PA-IR (*p* > 0.999) (Figure 4B). Necrosis was present in 7/7 PI-IR and 6/7 PA-IR animals and absent in all sham animals (sham vs. IR, *p* < 0.001; PI-IR vs. PA-IR, *p* = 1.000; Fisher’s exact test). Sinusoidal congestion was present in 7/7 PI-IR and 7/7 PA-IR animals, cytoplasmic vacuolization in 6/7 and 7/7, and inflammatory infiltrate in 7/7 and 6/7, respectively, whereas none of these lesions was observed in sham animals (Figure 4B).

### 3.3. Mitochondrial Respiration and H_2_O_2_ Emission

**Figure 5:**
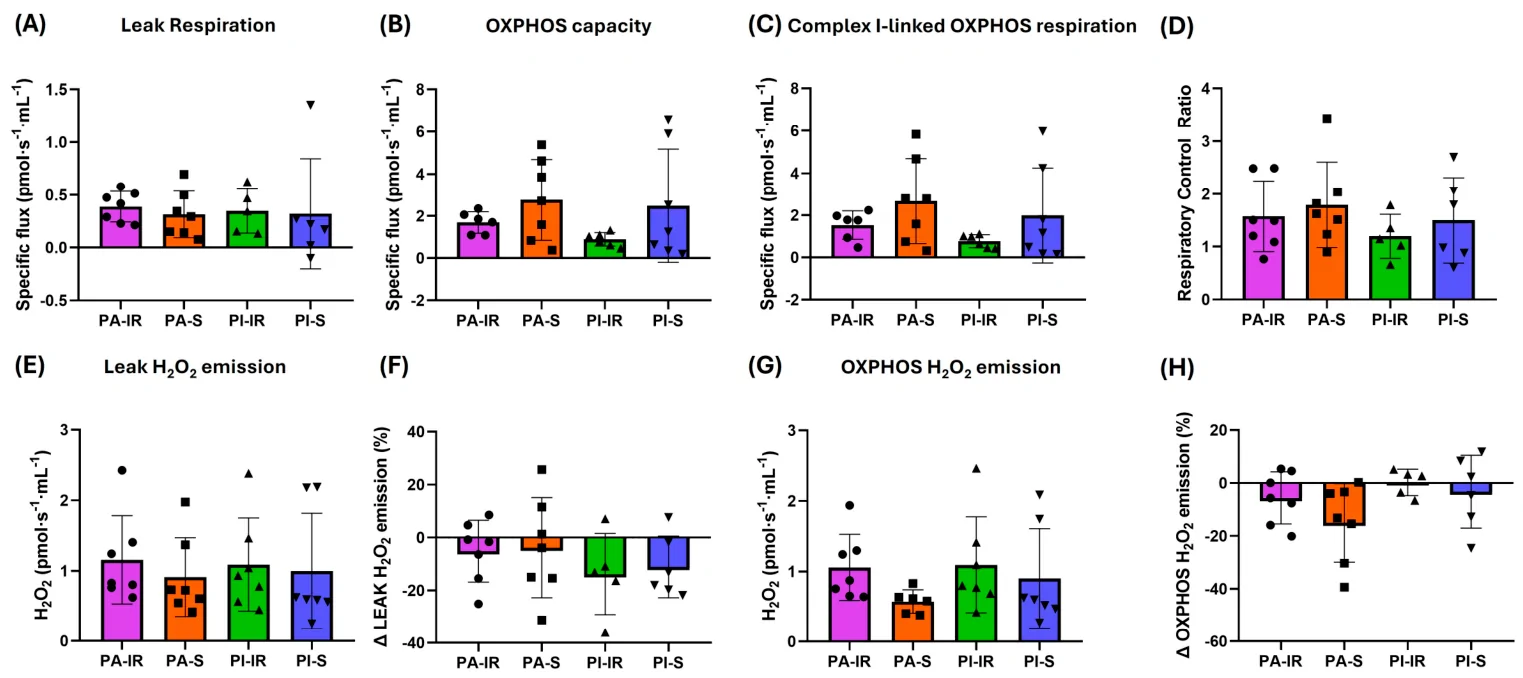
Mitochondrial respiration and hydrogen peroxide (H_2_O_2_) emission in liver homogenates across the four experimental groups (PA-IR, physically active ischemia–reperfusion; PA-S, physically active—sham; PI-IR, physically inactive ischemia–reperfusion; PI-S, physically inactive—sham). (**A**) Leak respiration; (**B**) OXPHOS capacity (ADP-stimulated respiration); (**C**) complex I-linked OXPHOS respiration (following pyruvate addition); (**D**) respiratory control ratio; (**E**) absolute H_2_O_2_ emission during leak respiration; (**F**) percentage change in leak H_2_O_2_ emission (Δ); (**G**) absolute H_2_O_2_ emission during OXPHOS; (**H**) percentage change in OXPHOS H_2_O_2_ emission (Δ). Oxygen flux and H_2_O_2_ emission are expressed as specific flux (pmol·s^−1^·mL^−1^). Bars represent mean ± SD, with individual values shown as symbols (*n* = 7 per group, except where outliers were excluded). The statistical analyses performed were: (**B**–**D**,**F**,**H**) one-way ANOVA test, followed by Tukey’s *post hoc* test or (**A**,**E**,**G**) Kruskal–Wallis test, followed by Dunn’s *post hoc* test.

High-resolution respirometry revealed no statistically significant effects of surgical procedure (PI-S+PA-S vs. PI-IR+PA-IR), activity (PA-S+PA-IR vs. PI-S+PI-IR), or their interaction on any mitochondrial respiration variable (Figure 5). Leak respiration, ADP-stimulated (State 3) and complex I-supported respiration, the respiratory control ratio (RCR), and mitochondrial H_2_O_2_ emission were all comparable across groups (all *p* > 0.05). The effects that came closest to significance were those related with complex I-supported State 3 respiration, which was lower after ischemia (PI-IR+PA-IR) than after sham surgery (PI-S+PA-S) [F(1,22) = 3.47, *p* = 0.076]. The JO_2_/H_2_O_2_ ratio during leak respiration was likewise lower after ischemia (PI-IR+PA-IR < PI-S+PA-S) [F(1,22) = 3.77, *p* = 0.065]. Activity on ADP-induced suppression of H_2_O_2_ emission was greater in physically active (PA-S+PA-IR) than in physically inactive groups (PI-S+PI-IR) [F(1,21) = 3.39, *p* = 0.080].

### 3.4. Liver Injury/Function

Systemic biochemical markers of liver function and injury revealed several significant differences related to surgical condition and physical activity. Albumin levels were significantly reduced in PI-IR+PA-IR (3.76 ± 0.08) compared to PI-S+PA-S (3.89 ± 0.07; *p* < 0.001), while no significant difference was found between PI-S+PI-IR (3.85 ± 0.09) and PA-S+PA-IR (3.81 ± 0.11). ALP activity was markedly higher in PI-IR+PA-IR (21.11 ± 16.30) compared to PI-S+PA-S (7.48 ± 2.33; *p* < 0.001). ALT activity was markedly higher in PI-IR+PA-IR (598.65 ± 353.26) compared to PI-S+PA-S (111.80 ± 51.85; *p* < 0.001), while no significant difference was observed between PI-S+PI-IR (327.96 ± 250.93) and PA-S+PA-IR (363.15 ± 426.63). AST levels were significantly higher in PI-IR+PA-IR (498.97 ± 192.89) compared to PI-S+PA-S (252.88 ± 76.96; *p* < 0.001), but no differences were found between PI-S+PI-IR (368.29 ± 151.97) and PA-S+PA-IR (374.23 ± 224.35). Bile acids were significantly elevated in PI-IR+PA-IR (24.46 ± 14.11) compared to PI-S+PA-S (13.99 ± 8.93; *p* = 0.018) and were also increased in PA-S+PA-IR (23.77 ± 15.34) compared to PI-S+PI-IR (13.94 ± 6.15; *p* = 0.018), with a significant interaction effect (*p* = 0.026). Total bilirubin was significantly elevated in PI-IR+PA-IR (0.08 ± 0.02) versus PI-S+PA-S (0.05 ± 0.01; *p* = 0.001) but remained similar between PI-S+PI-IR (0.06 ± 0.02) and PA-S+PA-IR (0.07 ± 0.02). For the remaining variables, both direct bilirubin (PI-S+PA-S: 0.07 ± 0.01; PI-IR+PA-IR: 0.089 ± 0.03; PI-S+PI-IR: 0.07 ± 0.01; PA-S+PA-IR: 0.09 ± 0.03) and amylase did not differ significantly (PI-S+PA-S: 739.25 ± 66.37; PI-IR+PA-IR: 724.01 ± 107.41; PI-S+PI-IR: 738.79 ± 92.59; PA-S+PA-IR: 724.46 ± 85.91) between groups (Table 1).

### 3.5. Apoptosis

**Figure 6:**
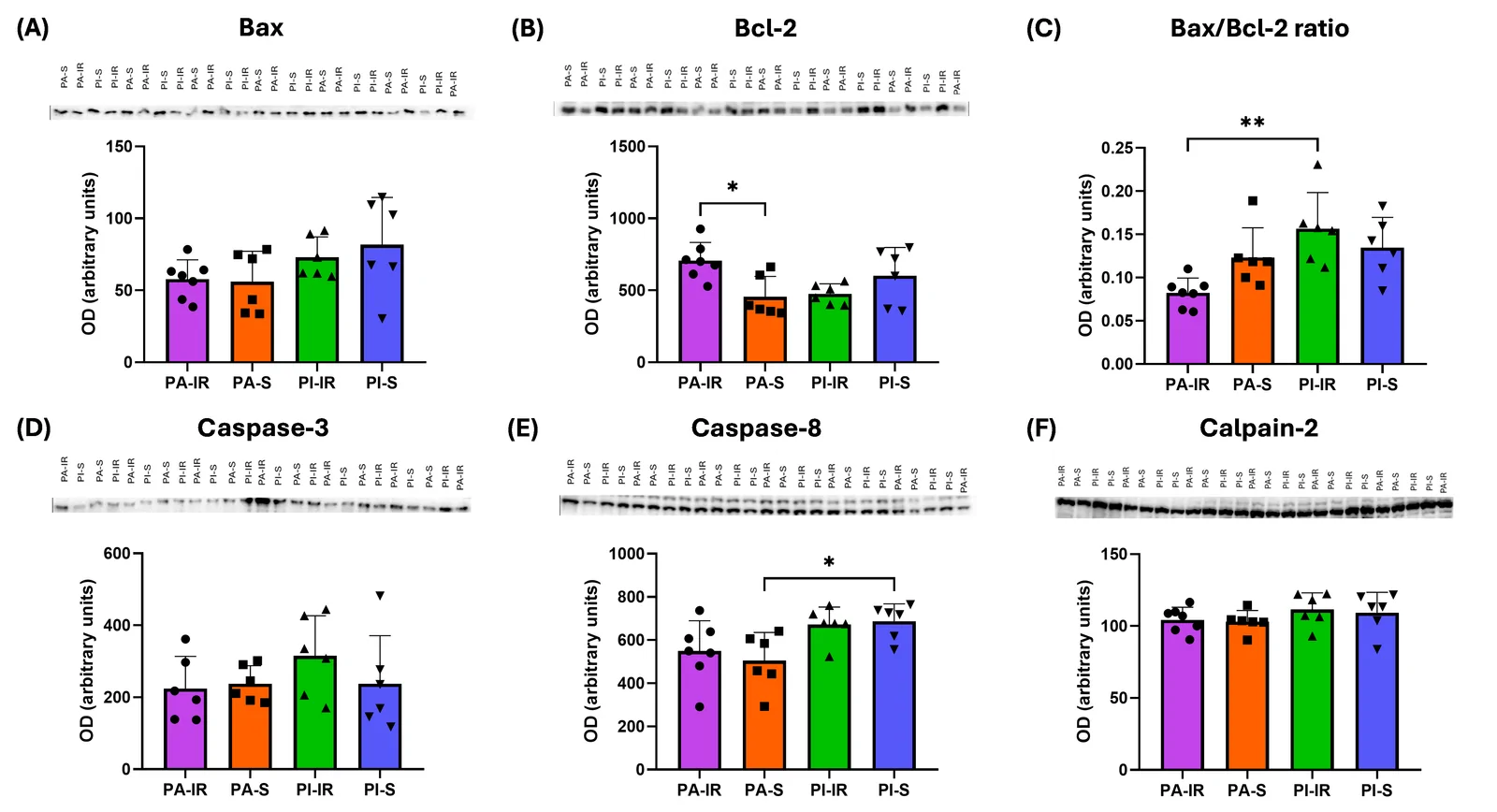
Apoptotic signaling in liver homogenates across the four experimental groups (PI-S, physically inactive—sham; PA-S, physically active—sham; PI-IR, physically inactive ischemia–reperfusion; PA-IR, physically active ischemia–reperfusion). (**A**) Bcl-2 associated X-protein (Bax) (21 kDa); (**B**) Bcl-2 (26 kDa); (**C**) Bax/Bcl-2 ratio; (**D**) caspase-3 (31 kDa); (**E**) caspase-8 (55 kDa); (**F**) calpain-2 (80 kDa). Protein levels are expressed as the optical density (OD) of the target band normalized to the Ponceau S total protein signal of the same lane (arbitrary units). Bars represent mean ± SD with individual values shown as symbols (*n* = 6–7 per group). The statistical analyses performed were: (**A**,**C**–**F**) one-way ANOVA test, followed by Tukey’s *post hoc* test or (**B**) Kruskal–Wallis test, followed by Dunn’s *post hoc* test (* *p* < 0.05; ** *p* < 0.01). Target-band strips and full Ponceau S-stained membranes are shown in Figure S1.

Bax/Bcl-2 ratio revealed significant differences between groups with PI-S+PI-IR exhibiting higher values (0.15 ± 0.04) compared to PA-S+PA-IR (0.10 ± 0.03; *p* = 0.004) (Table 1). Importantly, the Bax/Bcl-2 ratio was significantly higher in PI-IR compared to PA-IR animals (*p* = 0.0028) (Figure 6C). Nevertheless, there were no significant differences between groups according to surgical condition (PI-S+PA-S: 0.13 ± 0.03 vs. PI-IR+PA-IR: 0.12 ± 0.05). Furthermore, a significant interaction between surgical condition and activity status was observed (*p* = 0.026). Similarly, caspase-8 content was significantly elevated in PI-S+PI-IR (679.67 ± 77.26) compared to PA-S+PA-IR (528.53 ± 132.50; *p* = 0.003). However, values were comparable between PI-S+PA-S (595.68 ± 141.05) and PI-IR+PA-IR (606.06 ± 128.89) (Table 1). In contrast, no significant differences were observed for caspase-3 (PI-S+PA-S:237.77 ± 95.99 vs. PI-IR+PA-IR: 269.78 ± 107.54 and PI-S+PI-IR: 276.54 ± 123.96 vs. PA-S+PA-IR: 231.01 ± 69.59) or calpain-2 expression (PI-S+PA-S: 106.22 ± 11.29 vs. PI-IR+PA-IR: 107.68 ± 10.38 and PI-S+PI-IR: 110.47 ± 12.30 vs. PA-S+PA-IR: 103.75 ± 7.98) (Table 1).

### 3.6. Inflammation

**Figure 7:**
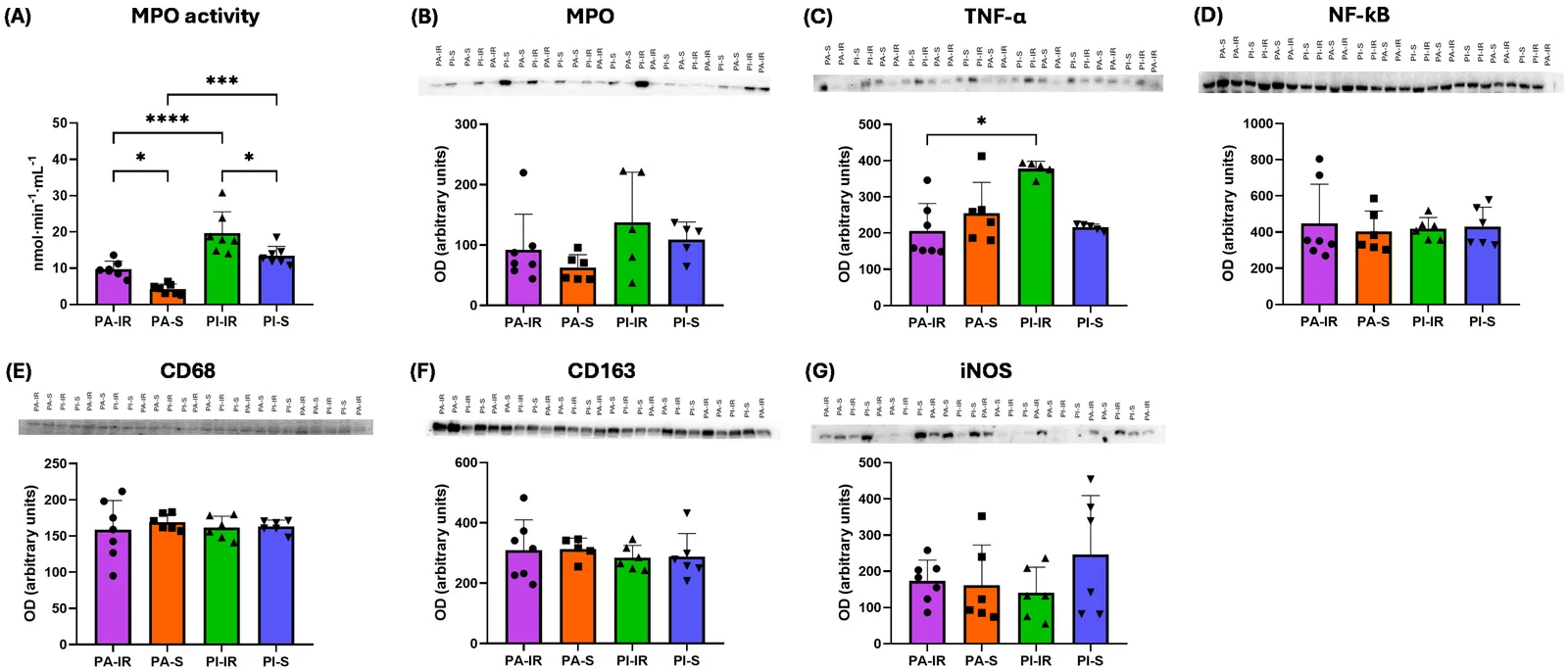
Inflammatory markers in liver homogenates across the four experimental groups (PI-S, physically inactive—sham; PA-S, physically active—sham; PI-IR, physically inactive ischemia–reperfusion; PA-IR, physically active ischemia–reperfusion). (**A**) Myeloperoxidase (MPO) activity; (**B**) MPO (59 kDa); (**C**) Tumour Necrosis Factor α (TNF-α) (25 kDa); (**D**) Nuclear factor kappa B p50 (NF-ĸB p50) (50 kDa); (**E**) CD68 (100 kDa); (**F**) CD163 (125 kDa); (**G**) Inducible nitric oxide synthase (iNOS) (131 kDa). MPO activity is expressed as nmol·min^−1^·mL^−1^. Protein levels are expressed as the optical density (OD) of the target band normalized to the Ponceau S total protein signal of the same lane (arbitrary units). Bars represent mean ± SD with individual values shown as symbols (*n* = 6–7 per group). The statistical analyses performed were: (**A**,**E**–**G**) one-way ANOVA test, followed by Tukey’s *post hoc* test or (**B**–**D**) Kruskal–Wallis test, followed by Dunn’s *post hoc* test (* *p* < 0.05; *** *p* < 0.001, **** *p* < 0.0001). Target-band strips and full Ponceau S-stained membranes are shown in Figure S1.

Regarding MPO activity, clear differences were observed between groups. MPO activity was significantly higher in PI-S+PI-IR (16.56 ± 5.44 nmol·min^−1^·mL^−1^) compared to PA-S+PA-IR (7.01 ± 3.35 nmol·min^−1^·mL^−1^; *p* < 0.001) as well as in PI-IR+PA-IR (14.72 ± 6.69 nmol·min^−1^·mL^−1^) compared to PI-S+PA-S (8.84 ± 5.16 nmol·min^−1^·mL^−1^; *p* < 0.001) (Table 1). Importantly, MPO activity was significantly lower in PA-IR (9.756 ± 2.175 nmol·min^−1^·mL^−1^) compared to PI-IR (19.683 ± 5.883 nmol·min^−1^·mL^−1^; *p* < 0.0001) (Figure 7A).

Tumor necrosis factor alpha (TNF-α) content was also lower in physically active groups (228.81 ± 80.63) compared to inactive counterparts (290.80 ± 82.90; *p* = 0.046) (Table 1) and, importantly, lower in PA-IR (206.057 ± 75.650) compared to PI-IR animals (349.435 ± 72.119; *p* < 0.0125) (Figure 7C). In contrast, no significant differences were observed for the levels of CD68 (PI-S+PA-S: 166.26 ± 10.02 vs. PI-IR+PA-IR: 159.74 ± 30.60; PI-S+PI-IR: 162.27 ± 12.33 vs. PA-S+PA-IR: 163.42 ± 30.18), CD163 (PI-S+PA-S: 331.06 ± 124.75 vs. PI-IR+PA-IR: 298.06 ± 77.00; PI-S+PI-IR: 285.98 ± 58.91 vs. PA-S+PA-IR: 339.66 ± 126.81), iNOS (PI-S+PA-S: 203.53 ± 140.67 vs. PI-IR+PA-IR: 158.34 ± 63.78; PI-S+PI-IR: 193.03 ± 132.42 vs. PA-S+PA-IR: 168.04 ± 82.88), NF-kB (PI-S+PA-S: 415.83 ± 106.94 vs. PI-IR+PA-IR: 434.05 ± 159.35; PI-S+PI-IR: 424.34 ± 83.91 vs. PA-S+PA-IR: 426.19 ± 172.03) and MPO (PI-S+PA-S: 122.07 ± 137.85 vs. PI-IR+PA-IR: 174.57 ± 238.84; PI-S+PI-IR: 226.31 ± 260.77 vs. PA-S+PA-IR: 78.35 ± 46.70) (Table 1).

### 3.7. Oxidative Stress

**Figure 8:**
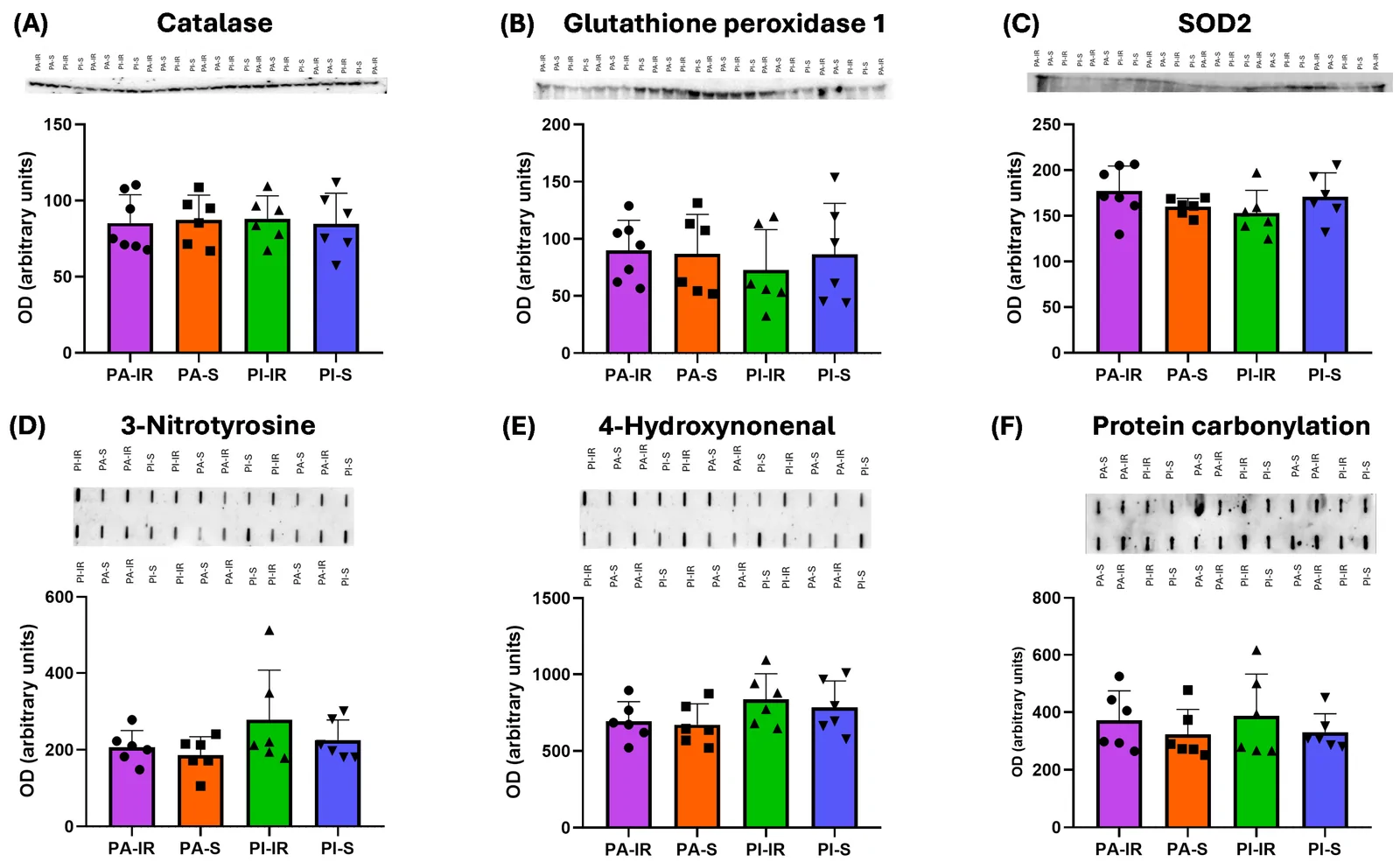
Oxidative stress markers in liver homogenates across the four experimental groups (PI-S, physically inactive—sham; PA-S, physically active—sham; PI-IR, physically inactive ischemia–reperfusion; PA-IR, physically active ischemia–reperfusion). (**A**) Catalase (60 kDa); (**B**) glutathione peroxidase 1 (55 kDa); (**C**) Superoxide Dismutase 2 (SOD2) (24 kDa); (**D**) 3-nitrotyrosine (3-NT); (**E**) 4-hydroxynonenal (4-HNE); (**F**) protein carbonylation. Protein levels are expressed as the optical density (OD) of the target band normalized to the Ponceau S total protein signal of the same lane (arbitrary units). Bars represent mean ± SD with individual values shown as symbols (*n* = 6–7 per group). The statistical analyses performed were: (**A**–**C**,**E**,**F**) one-way ANOVA test, followed by Tukey’s *post hoc* test or (**D**) Kruskal–Wallis test, followed by Dunn’s *post hoc* test. Target-band strips and full Ponceau S-stained membranes are shown in Figure S1.

Oxidative stress markers were not significantly different between groups (Figure 8). In fact, no significant differences were observed for catalase (PI-S+PA-S: 86.09 ± 17.42 vs. PI-IR+PA-IR: 86.55 ± 16.35; PI-S+PI-IR: 86.42 ± 16.98 vs. PA-S+PA-IR: 86.25 ± 16.77), glutathione peroxidase 1 (PI-S+PA-S: 89.71 ± 37.96 vs. PI-IR+PA-IR: 81.85 ± 30.88; PI-S+PI-IR: 79.64 ± 39.00 vs. PA-S+PA-IR: 88.37 ± 29.21), 3-NT (PI-S+PA-S: 205.82 ± 51.66 vs. PI-IR+PA-IR: 242.29 ± 99.42; PI-S+PI-IR: 251.50 ± 98.14 vs. PA-S+PA-IR: 196.61 ± 44.78), 4-HNE (PI-S+PA-S: 728.29 ± 158.62 vs. PI-IR+PA-IR: 764.44 ± 160.71; PI-S+PI-IR: 810.00 ± 165.10 vs. PA-S+PA-IR: 682.73 ± 124.93), protein carbonylation (PI-S+PA-S: 326.86 ± 72.65 vs. PI-IR+PA-IR: 379.50 ± 120.38; PI-S+PI-IR: 359.11 ± 111.08 vs. PA-S+PA-IR: 347.26 ± 94.16) and SOD2 (PI-S+PA-S: 165.35 ± 19.54 vs. PI-IR+PA-IR: 165.90 ± 28.17; PI-S+PI-IR: 191.90 ± 26.03 vs. PA-S+PA-IR: 169.09 ± 22.26). No statistically significant main effects or interactions were observed for any of the oxidative stress markers (Table 1).

### 3.8. Endothelial Dysfunction

**Figure 9:**
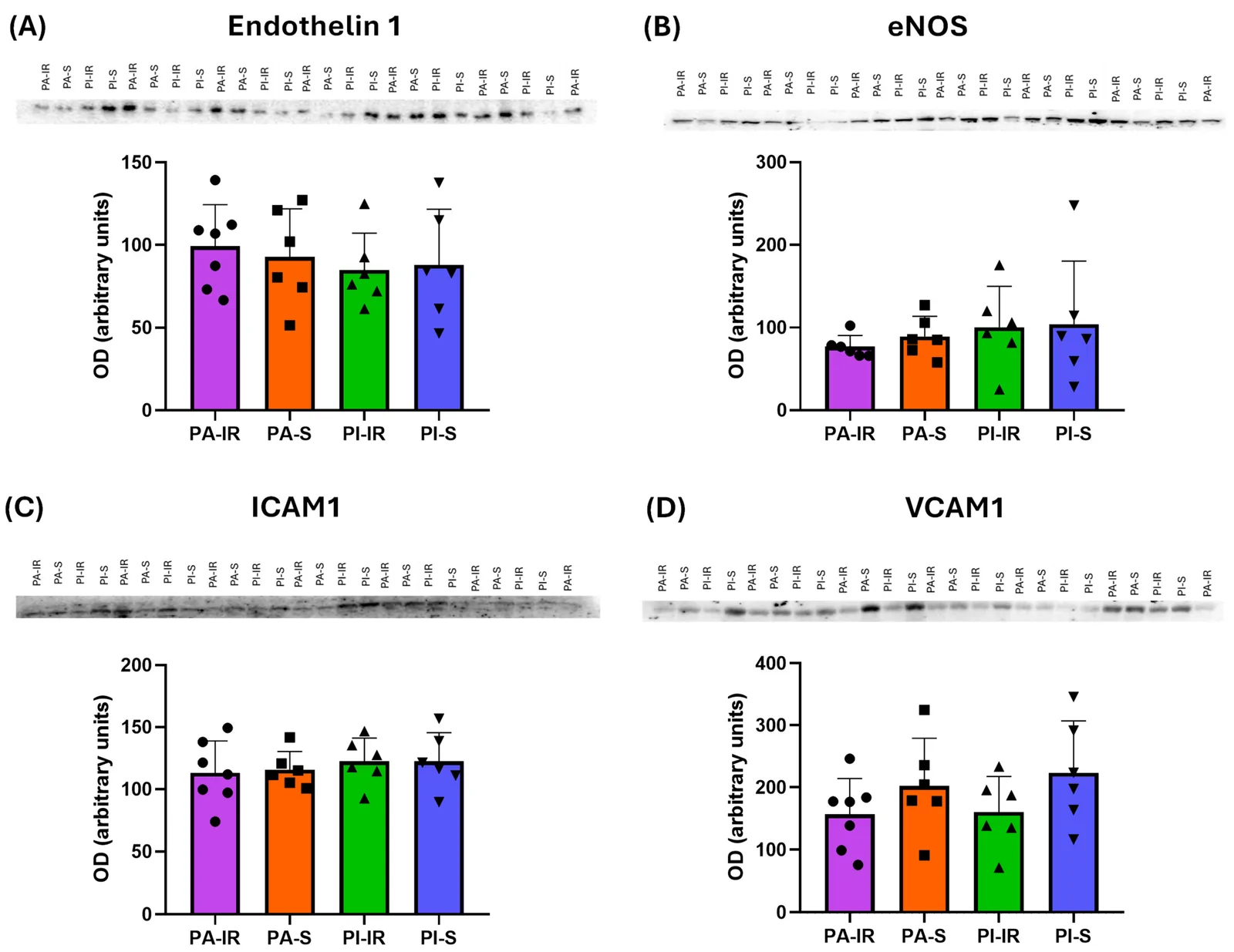
Endothelial dysfunction markers in liver homogenates across the four experimental groups (PI-S, physically inactive—sham; PA-S, physically active—sham; PI-IR, physically inactive ischemia–reperfusion; PA-IR, physically active ischemia–reperfusion). (**A**) Endothelin-1 (ET-1) (24 kDa); (**B**) Endothelial nitric oxide synthase (eNOS) (133 kDa); (**C**) Intercellular Adhesion Molecule 1 (ICAM1) (57 kDa); (**D**) Vascular Cell Adhesion Molecule-1 (VCAM1) (81 kDa). Protein levels are expressed as the optical density (OD) of the target band normalized to the Ponceau S total protein signal of the same lane (arbitrary units). Bars represent mean ± SD with individual values shown as symbols (*n* = 6–7 per group). The statistical analyses performed were: (**A**–**D**) one-way ANOVA test, followed by Tukey’s *post hoc* test. Target-band strips and full Ponceau S-stained membranes are shown in Figure S1.

Endothelial dysfunction markers were not statistically different between groups (Figure 9). Mean levels of Endothelin 1 were 90.38 ± 30.15 in PI-S+PA-S, 92.67 ± 24.03 in PI-IR+PA-IR, 86.50 ± 27.21 in PI-S+PI-IR and 96.26 ± 26.17 in PA-S+PA-IR. eNOS level was 96.66 ± 54.47 for PI-S+PA-S, 92.80 ± 38.11 for PI-IR+PA-IR, 102.32 ± 61.19 for PI-S+PI-IR, and 87.58 ± 25.20 for PA-S+PA-IR. Regarding ICAM1, levels were 119.10 ± 18.77 for PI-S+PA-S, 117.45 ± 22.37 for PI-IR+PA-IR, 122.43 ± 20.09 for PI-S+PI-IR and 114.38 ± 20.52 for PA-S+PA-IR. For VCAM1, mean levels were 212.69 ± 77.56 for PI-S+PA-S, 158.56 ± 54.81 for PI-IR+PA-IR, 191.81 ± 75.87 for PI-S+PI-IR and 177.83 ± 68.29 for PA-S+PA-IR. No statistically significant differences were observed between any of the experimental groups regarding all variables of endothelial dysfunction (Table 1).

### 3.9. Mitochondrial Biogenesis and Dynamics

**Figure 10:**
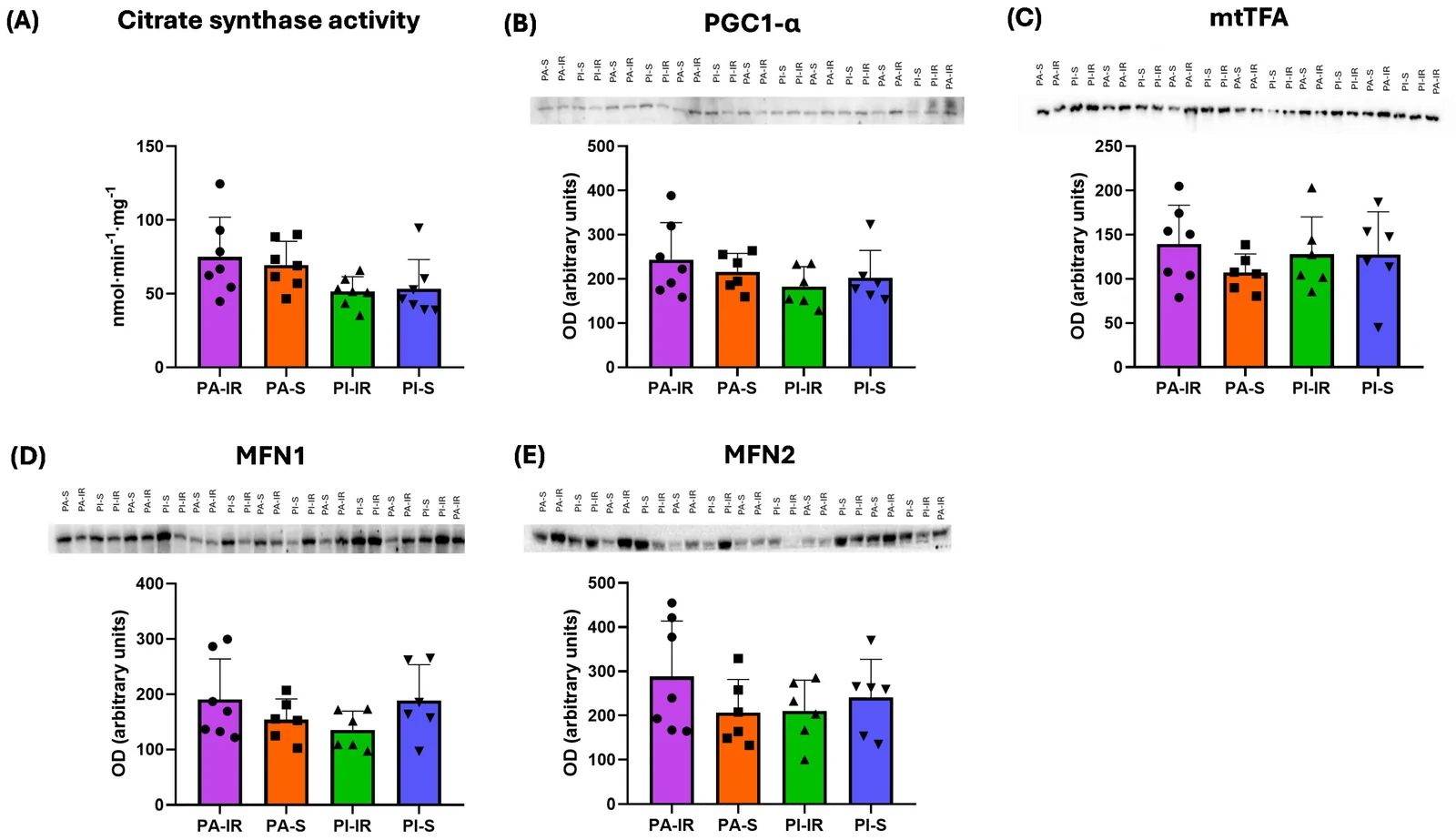
Mitochondrial content, biogenesis and fusion markers in liver homogenates across the four experimental groups (PI-S, physically inactive—sham; PA-S, physically active—sham; PI-IR, physically inactive ischemia–reperfusion; PA-IR, physically active ischemia–reperfusion). (**A**) Citrate synthase (CS) activity; (**B**) Peroxisome proliferator-activated receptor gamma coactivator 1-alpha (PGC1-α) (92 kDa); (**C**) Mitochondrial transcription factor A (mtTFA) (25 kDa); (**D**) Mitofusin 1 (MFN1) (86 kDa); (**E**) Mitofusin 2 (MFN2) (86 kDa). CS activity is expressed as nmol·min^−1^·mg^−1^. Protein levels are expressed as the optical density (OD) of the target band normalized to the Ponceau S total protein signal of the same lane (arbitrary units). Bars represent mean ± SD with individual values shown as symbols (*n* = 6–7 per group). The statistical analyses performed were: (**C**–**E**) one-way ANOVA test, followed by Tukey’s *post hoc* test or (**A**,**B**) Kruskal–Wallis test, followed by Dunn’s *post hoc* test. Target-band strips and full Ponceau S-stained membranes are shown in Figure S1.

Mitochondrial dysfunction markers showed no statistically significant differences between groups (Figure 10), except for CS activity, which was significantly higher in physically active groups (PA-S+PA-IR: 72.21 ± 21.51 nmol·min^−1^·mg^−1^) compared to inactive counterparts (PI-S+PI-IR: 52.48 ± 15.04 nmol·min^−1^·mg^−1^; *p* = 0.012, Table 1). Regarding the effect of IR, CS activity was similar between groups that underwent IR (PI-IR+PA-IR: 63.22 ± 23.03 nmol·min^−1^·mg^−1^) and sham intervention (PI-S+PA-S: 61.48 ± 19.14 nmol·min^−1^·mg^−1^). No statistically significant differences were also observed in mitochondrial fusion proteins or transcription factors. Specifically, MFN1 levels were 161.80 ± 56.92 in PI-S+PI-IR, 173.51 ± 60.48 in PA-S+PA-IR, 171.11 ± 53.88 PI-S+PA-S and 164.92 ± 63.38 in PI-IR+PA-IR. MFN2 levels were 225.82 ± 76.30 for PI-S+PI-IR, 250.77 ± 109.75 for PA-S+PA-IR, 223.89 ± 79.07 for PI-S+PA-S and 252.54 ± 107.46 for PI-IR+PA-IR. mtTFA levels were 127.75 ± 43.30 in PI-S+PI-IR, 124.44 ± 37.90 in PA-S+PA-IR, 117.43 ± 37.06 in PI-S+PA-S and 133.96 ± 41.94 in PI-IR+PA-IR. Finally, PGC1-α levels were 192.45 ± 52.66 in PI-S+PI-IR, 230.76 ± 66.66 in PA-S+PA-IR, 208.97 ± 51.10 in PI-S+PA-S and 215.51 ± 73.03 in PI-IR+PA-IR. No statistically significant differences were found between groups for any of these mitochondrial dysfunction variables (Table 1).

### 3.10. Systemic Markers

None of systemic metabolic markers assessed reached statistically significant differences between the experimental groups. Specifically, creatinine concentration was similar between PI-S+PA-S (0.26 ± 0.05), PI-IR+PA-IR (0.28 ± 0.04), PI-S+PI-IR (0.28 ± 0.04) and PA-S+PA-IR (0.27 ± 0.06). CK concentration was also similar across groups (PI-S+PA-S: 227.81 ± 140.61, PI-IR+PA-IR: 186.11 ± 44.24, PI-S+PI-IR: 233.22 ± 140.16 and PA-S+PA-IR: 180.71 ± 39.18). Also, no differences were observed in glucose (PI-S+PA-S: 235.85 ± 41.55, PI-IR+PA-IR: 205.55 ± 27.90, PI-S+PI-IR: 221.42 ± 36.78 and PA-S+PA-IR: 219.98 ± 40.56) or lactate concentrations (PI-S+PA-S: 27.05 ± 5.95, PI-IR+PA-IR: 28.73 ± 5.73, PI-S+PI-IR: 27.58 ± 5.77 and PA-S+PA-IR: 28.21 ± 6.02 mg.dL^−1^) (Table 1).

## 4. Discussion

To our knowledge, this is the first study to assess whether long-term physical inactivity modifies the hepatic response to ischemia–reperfusion (IR) injury in adult male Wistar rats using a factorial design that dissociates housing condition from surgical procedure. The major findings of this study were that (1) hepatic IRI was successfully induced, as demonstrated by hepatocellular and hepatobiliary dysfunction; (2) physical inactivity did not aggravate the primary hepatocellular injury, as reflected by comparable circulating transaminase responses between physically inactive and physically active animals; (3) physically inactive animals exhibited an exaggerated inflammatory and an upregulation of pro-apoptotic signaling in response to reperfusion, evidenced by higher MPO activity, TNF-α levels and Bax/Bcl-2 ratio; (4) these differences were not explained by detectable alterations in oxidative stress, endothelial markers, mitochondrial respiration, mitochondrial H_2_O_2_ emission, or the content of proteins involved in mitochondrial biogenesis and fusion, suggesting that these pathways were unlikely to account for the observed phenotype at the 24 h reperfusion time point; and (5) caspase-8 and CS activity differed according to physical activity condition, but without a significant interaction, indicating a constitutive rather than an IR-dependent effect.

Food intake was consistently higher in the physically active groups throughout the 25-week intervention. This increase remained within the physiological range reported for adult male Wistar rats under ad libitum feeding conditions [31] and is consistent with the compensatory increase in energy intake commonly observed in rodents with voluntary wheel running [39]. Despite the higher food intake, physically active animals exhibited body weights comparable to physically inactive counterparts, in line with their lower energy expenditure. Importantly, running wheel distance was comparable between PA-S and PA-IR animals, indicating that both groups were exposed to a similar activity workload. Running distances were also consistent with those previously reported in physically active rats with free access to running wheels [40].

Hepatic IR resulted in a marked increase in circulating liver transaminases, confirming the successful induction of hepatocellular injury. Histological examination of H&E-stained liver sections corroborated this biochemical evidence, revealing focal hepatocyte coagulative necrosis and sinusoidal congestion in IR animals compared with preserved lobular architecture in sham controls and further supporting successful induction of hepatic IRI in the present model. Semi-quantitative scoring confirmed this pattern: the Suzuki score and the incidence of each lesion increased after IR but did not differ between PI-IR and PA-IR animals, in agreement with the comparable transaminase responses. ALT and AST are released following disruption of hepatocyte membrane integrity and therefore reflect the extent of acute hepatocellular damage after ischemia–reperfusion [41]. Importantly, transaminase increase was comparable between groups, indicating that long-term physical inactivity did not aggravate hepatocellular injury induced by the ischemic insult. These findings partially differ from those of Yazdani et al. [41], who reported that, in mice, four weeks of treadmill exercise (12.5 m·min^−1^) preconditioning attenuated the post-ischemic rise in both ALT and AST, together with reduced necrosis and inflammation. Several methodological differences may account for this discrepancy. Specifically, their protocol employed a longer ischemic period of 60 min [41] compared to our 30 min, as well as a structured treadmill exercise regimen consisting of 60 min of daily running for 5 consecutive days/week [41], whereas our model relied on spontaneous voluntary physical activity driven by the animals natural running behavior. In addition, hepatocellular injury evolves dynamically during reperfusion. While the initial ischemic insult directly compromises hepatocyte integrity, subsequent injury is amplified by sterile inflammatory mechanisms involving Kupffer cell activation, cytokine release, and neutrophil recruitment [42]. Because transaminases were measured only after 24 h of reperfusion, they represent the cumulative consequence of both the primary ischemic insult and the subsequent inflammatory phase. Nevertheless, the comparable ALT and AST responses observed in physically inactive and active animals indicate that physical inactivity did not exacerbate the initial hepatocellular injury.

Hepatobiliary function was also affected in the IRI model, with increased ALP, bile acids and total bilirubin, which is in line with reports of early accumulation of circulating bile salts and reduced bile flow in ischemic liver tissue [43] and with the association between bile acid and bilirubin accumulation and histological injury severity [44]. A significant procedure × activity interaction was additionally observed for ALP (*p* = 0.021) and bile acids (*p* = 0.026), with peak values occurring in the PA-IR group. At first glance, this finding could suggest more severe hepatobiliary injury in physically active animals. However, such an interpretation appears unlikely, as circulating ALT and AST were comparable between the inactive and active ischemic groups, indicating that the higher cholestatic markers were not accompanied by greater hepatocellular damage. Instead, these findings suggest that habitual physical activity may have modified the hepatobiliary response to IR rather than the severity of the injury itself. One possible explanation is that physically active animals exhibited a more effective adaptive response aimed at limiting intracellular bile acid accumulation. During cholestatic stress, hepatocytes upregulate basolateral export transporters such as MRP3, MRP4 and OSTα/β, promoting the efflux of bile acids into the systemic circulation while reducing their intracellular retention [45], an adaptive response shown to reduce hepatic injury [46]. If this response was more pronounced in physically active animals, it could account for the higher circulating bile acid concentrations despite similar hepatocellular injury. A similar interpretation may apply to ALP, although its increase is more difficult to attribute to a single mechanism because circulating ALP reflects both increased hepatic synthesis and the release of membrane-bound enzyme secondary to bile salt-induced membrane injury [47]. This interpretation nonetheless remains speculative, since only total bile acids were quantified, so neither the species responsible for the increase nor the underlying molecular mechanisms can be identified.

Albumin was significantly lower in the ischemic groups (*p* < 0.001). Given the relatively small magnitude of this reduction (approximately 3%) and the plasma half-life of albumin [48] it is unlikely that this finding reflects impaired hepatic albumin synthesis within the 24 h reperfusion period. The decrease in albumin is more likely attributable to the acute-phase response, in which increased vascular permeability and redistribution of albumin from the intravascular to the interstitial compartment contribute to lower circulating concentrations during acute inflammation [49]. Consequently, albumin concentration does not seem to be able to be interpreted as a reliable indicator of hepatic synthetic function under the present experimental conditions.

The inflammatory response represented the principal pathway through which physical inactivity influenced hepatic ischemia–reperfusion injury. Neutrophil infiltration is a hallmark of the secondary phase of hepatic IR injury, during which activated neutrophils amplify tissue damage through the release of reactive oxygen species (ROS), proteases and pro-inflammatory mediators [50,51]. In the present study, MPO activity was significantly affected by both the surgical procedure and physical activity condition (*p* < 0.001), with a significant interaction. These findings are consistent with those of Mohammadkhani et al. [52], who demonstrated, in a myocardial IR model, that different exercise modalities, including endurance, resistance, and concurrent training, reduced MPO activity following IRI. In line with this finding, our results showed that physically inactive animals exhibited significantly higher MPO activity compared to physically active counterparts, suggesting that inactivity favors an exaggerated neutrophil-mediated inflammatory response. This interpretation is further supported by TNF-α results. As one of the earliest cytokines released by activated Kupffer cells [53], TNF-α orchestrates the hepatic inflammatory cascade by promoting endothelial activation, leukocyte recruitment and cytokine amplification [54]. In the present work, TNF-α increased in PI-IR animals (349.4 ± 72.1) but not in PA-IR animals (206.1 ± 75.7), with significant interaction (*p* = 0.008). Similar reductions in TNF-α following exercise preconditioning were described by Lai et al. [55] in a myocardial IR model. In fact, they demonstrated that four weeks of high-intensity interval training (HIIT) prior to myocardial ischemia–reperfusion significantly attenuated circulating TNF-α levels compared with sedentary rats. Notably, although our animals engaged in spontaneous voluntary wheel running rather than a structured exercise intervention, the free-wheel running model has been shown to reproduce activity patterns comparable to those observed during HIIT [56], supporting the relevance of this comparison. Together with the MPO findings, these results indicate that long-term physical inactivity promotes a more pronounced pro-inflammatory response during hepatic reperfusion.

Interestingly, MPO protein content did not parallel with the changes observed in MPO activity. While MPO activity exhibited a significant procedure × activity interaction, MPO protein abundance remained unchanged, suggesting that physical inactivity primarily influenced neutrophil activation or degranulation rather than the absolute number of infiltrating neutrophils. Likewise, neither CD68 nor CD163 levels differed between groups at the 24 h reperfusion time point. However, this finding should be interpreted cautiously, as macrophages contribute not only to the initiation of inflammation but also to its subsequent resolution and tissue repair, with their phenotypic profile evolving dynamically throughout reperfusion [57]. In this regard, Liu et al. [57] demonstrated that exercise-induced hypertrophic preconditioning, induced through 21 days of swimming training (5 days/week) followed by a one-week detraining period before IR, promoted a shift from pro-inflammatory M1 macrophages to anti-inflammatory M2 macrophages 24 h after reperfusion. Likewise, the experimental model differed from ours, as that study was performed in C57BL/6 mice and evaluated myocardial, under a structured swimming protocol rather than voluntary activity [57]. Although performed in a different experimental model, these findings support the concept that regular physical activity modulates the inflammatory response at multiple levels. Overall, our data indicates that the detrimental effect of physical inactivity on hepatic IRI is primarily associated with an enhanced neutrophil-driven inflammatory response, whereas potential effects on macrophage populations were not detectable under the experimental conditions employed.

Findings on apoptotic cell death are consistent with this interpretation but require careful distinction. The Bax/Bcl-2 ratio, a key regulator of mitochondrial outer membrane permeabilization and the intrinsic apoptotic pathway [58] was highest in PI-IR animals (0.157 ± 0.042) and lowest in PA-IR animals (0.082 ± 0.017), with a significant interaction (*p* = 0.026). In line with this finding, Aboutaleb et al. [59] demonstrated, in a rat model, that four weeks of progressive treadmill training (5 days/week, 35–50 min with progressively increasing inclines from 0° to 15°, at 18 m·min^−1^) significantly attenuated brain ischemia-induced Bax/Bcl-2 ratio increase and reduced apoptotic cell death. The parallel is defensible because the Bax/Bcl-2 axis governs a mitochondrial apoptotic mechanism that is broadly conserved across tissues, unlike antioxidant and endothelial responses, which are markedly organ-dependent. Although the magnitude of the response differed between models, both studies indicate that prior physical activity shifts the balance of Bcl-2 family proteins towards a less pro-apoptotic phenotype, thereby limiting activation of the intrinsic apoptotic pathway following ischemic injury.

Caspase-8, the initiator protease of the extrinsic apoptotic pathway downstream of TNF receptor activation [60,61], was significantly lower in physically active animals, but neither an effect of IR nor a procedure × activity interaction was detected. In pairwise comparisons, this difference reached significance between PA-S and PI-S but not between PA-IR and PI-IR, possibly because sham surgery itself induces inflammatory and stress responses [62,63], which physical activity may attenuate, whereas the greater IR insult may have masked this effect. Importantly, this reduction in caspase-8 was not accompanied by changes in the effector protease caspase-3, indicating that lower initiator-caspase levels did not translate into effector-caspase activation. The dissociation between caspase-8 and the Bax/Bcl-2 ratio suggests that physical activity is associated with a constitutively lower pro-apoptotic signaling tone through the extrinsic (death receptor-mediated) pathway, independent of reperfusion, whereas the intrinsic (mitochondrial) pathway showed a distinct, reperfusion-related pattern. Physical inactivity may therefore be associated with a greater propensity for pro-apoptotic signaling, although the absence of an interaction precludes attributing this difference specifically to ischemia–reperfusion.

The lack of an IR effect on caspase-8 differs from the findings of Fauconnier et al. [64], who reported increased myocardial caspase-8 activity following IR. Three methodological differences may account for this apparent discrepancy. First, their study examined a myocardial rather than a hepatic model and identified a heart-specific TNF-α-caspase-8 pathway associated with altered RyR2 function, calcium handling and ventricular injury. Second, caspase-8 activity was assessed at multiple reperfusion time points and peaked at 6 h before returning towards baseline by 24 h, whereas liver samples in the present study were collected only at 24 h, so a transient early activation may have been missed. Third, and perhaps most importantly, Fauconnier et al. [64] measured caspase-8 enzymatic activity, whereas we assessed protein levels. Nonetheless, caspase-3 abundance was also unchanged. Since caspase-3 is the downstream effector of caspase-8, an earlier caspase-8 activation would be expected to leave a detectable effector signature at 24 h. In fact, its absence provides independent evidence that the extrinsic pathway did not proceed to effector-caspase activation within this time frame, rather than simply reflecting a missed early time point. Furthermore, no significant differences were found in calpain-2 expression, which contributes to apoptosis through cleavage of Bax and Bid [65], suggesting that the changes observed in upstream apoptotic signaling did not translate into measurable differences at the effector phase by this time point.

Given the central role of mitochondria in both oxidative stress and intrinsic apoptosis, as well as in the pathophysiology of IRI [66], we investigated whether mitochondrial adaptations could be divergent between physically inactive and active animals. CS activity, a classical marker of mitochondrial content [67], was significantly higher in physically active animals, which agrees with the well-documented mitochondrial adaptations induced by regular physical activity in skeletal muscle [68]. However, the interaction was not significant, indicating that the higher CS activity in physically active animals represents a pre-existing mitochondrial adaptation associated with habitual physical activity rather than a differential response to IR. Contra-intuitively; this increase in mitochondrial content was not accompanied by changes in PGC1-α, mtTFA, MFN1 or MFN2 expression, nor by any difference in mitochondrial respiration variables (leak, OXPHOS capacity, complex I-linked respiration and respiratory control ratio) or H_2_O_2_ emission. A possible explanation for the absence of differences in mitochondrial biogenesis markers despite the higher CS activity may reflect the transient nature of the molecular signals regulating mitochondrial adaptation. PGC1-α and related transcriptional regulators are activated acutely following individual bouts of exercise, with PGC-1α mRNA peaking within 2 h after exercise and returning to basal levels within 24 h in human skeletal muscle [69]. Furthermore, enzymatic activity is more transient than protein levels, being influenced by post-translational modifications and substrate availability [70]. In the liver, elevated CS activity may also reflect a greater reliance on the TCA cycle for its amphibolic, anabolic role rather than mitochondrial density alone [71].

In addition, ex vivo respirometry performed under saturating substrate conditions may not detect functional advantages that become apparent only under the metabolic and oxidative stress associated with reperfusion *in vivo* [72]. Although additional mechanisms may contribute, our findings suggest that physical activity increased mitochondrial content without producing detectable changes in basal intrinsic mitochondrial respiratory function.

Oxidative stress is widely considered a central mechanism of hepatic IRI [73], and many of the beneficial effects of exercise in this regard have been attributed to enhanced antioxidant capacity [74]. Surprisingly, however, neither antioxidant enzymes (catalase, glutathione peroxidase 1 and SOD2) nor oxidative damage markers (3-NT, 4-HNE and protein carbonyls) differed between physically inactive and physically active animals. One possible explanation is the timing of tissue collection, as oxidative stress is believed to peak during the early stages of reperfusion and may have largely resolved by the 24 h time point examined here [75]. Although enhanced antioxidant defenses are frequently proposed as a major mediator of exercise-induced protection, the absence of detectable differences in antioxidant markers in the present study suggests that other mechanisms, particularly the modulation of inflammatory and apoptotic signaling, may have contributed more substantially to the observed effects. It should also be noted that antioxidant enzyme activity can be regulated independently of protein abundance, such that functional adaptations may not necessarily be reflected by changes in protein levels.

Endothelial markers were assessed to determine whether hepatic IR could have induced local vascular alterations that could also be modulated by physical (in)activity. A similar pattern was observed for the endothelial markers, endothelin-1 (ET-1), eNOS, ICAM1 and VCAM1, since none differed significantly between groups. These findings contrast with the well-established view that regular physical activity preserves endothelial function and attenuates endothelial activation [76]. Endothelial-specific alterations may have been masked because protein expression was quantified in whole-tissue homogenates rather than in isolated liver sinusoidal endothelial cells. Since the molecular signal obtained from whole liver samples is largely dominated by hepatocytes, subtle changes occurring in the sinusoidal endothelium may not have been detected [77]. Overall, we cannot exclude the effect of habitual physical activity on the hepatic endothelium, but under the present experimental conditions no such effect was detectable at the whole-tissue level.

Beyond hepatocellular injury, IRI may compromise the systemic functions of the liver. The liver synthesizes most coagulation factors and natural anticoagulants, and coagulopathy is a hallmark of hepatic failure [78]. It is also the main source of circulating insulin-like growth factor-1 [79] and, to a lesser extent in adults, of erythropoietin [80], and it produces angiotensinogen, the substrate of the renin–angiotensin system and a determinant of blood pressure regulation [81]. In addition, the liver plays a central role in glucose, lipid and nitrogen metabolism, including albumin and urea synthesis and detoxification [82]. Consequently, extensive hepatocellular injury may result in coagulopathy, hemodynamic instability, metabolic disturbances and encephalopathy [83]. In the present study, however, the absence of differences in circulating creatinine, CK, glucose and lactate indicates that hepatic IR did not induce detectable extrahepatic organ dysfunction or generalized metabolic disturbance under the present experimental conditions. This observation strengthens the interpretation that the differences observed in inflammatory and apoptotic signaling primarily reflect local hepatic responses rather than systemic confounding factors. Moreover, because AST can originate from skeletal muscle as well as the liver [84], the unchanged CK concentrations support the interpretation that the increase in circulating AST predominantly reflected hepatocellular injury.

Several limitations should be considered. First, hepatic injury was assessed exclusively at 24 h after reperfusion, capturing a single point of a highly dynamic process in which oxidative, endothelial, mitochondrial, inflammatory and apoptotic events follow distinct temporal kinetics [85]. Transient adaptations associated with habitual physical activity may therefore have fallen outside this window. Serial sampling across reperfusion, combined with histopathological and functional assessments, would have allowed these temporal mechanisms to be analyzed with higher granularity. Second, although cage wheel running is a biologically relevant model of voluntary physical activity, self-selected running introduces high inter-individual variability in volume and intensity, which increases data dispersion and reduces the power to detect modest biological effects. Combining voluntary running with standardized protocols of defined intensity and duration would allow establishing dose–response relationships between exercise exposure and hepatic protection [86]. Third, the use of young, metabolically healthy animals, while limiting biological variability, restricts translational applicability. Clinically, hepatic IRI most often affects older individuals with metabolic dysfunction-associated steatotic liver disease, obesity and insulin resistance [87], conditions that alter mitochondrial bioenergetics, redox homeostasis and regenerative capacity [88]. Whether habitual physical activity confers comparable protection in these settings remains unknown and addressing this question in aged or steatotic models is a necessary step towards translation. Finally, only male rats were used to avoid the additional variability associated with the estrous cycle, which influences both voluntary wheel running [89] and hepatic susceptibility to IRI [90]. Future studies including female cohorts are needed to strengthen the translational relevance of these findings.

## 5. Conclusions

Long-term physical inactivity did not increase the magnitude of the primary hepatocellular injury induced by hepatic IR, since circulating transaminases rose comparably in physically inactive and active animals. Physically inactive animals subjected to IR nonetheless displayed the highest MPO activity, TNF-α expression and Bax/Bcl-2 ratio, and the significant procedure × activity interactions detected for these markers indicate that the two housing conditions lead to different responses to the same insult rather than simply differing at baseline. That this interaction was detected for MPO activity but not for MPO protein abundance further suggests that inactivity influenced neutrophil functional activation rather than neutrophil recruitment itself. By contrast, caspase-8 and CS activity differed with physical activity conditions in the absence of significant interaction, consistent with a constitutive adaptation to physical activity.

From a mechanistic standpoint, no differences were found in oxidative stress, endothelial function, mitochondrial respiration or mitochondrial biogenesis and fusion markers, suggesting that the effects observed here relate more closely to inflammatory and apoptotic pathways than to antioxidant, endothelial or bioenergetic mechanisms. These findings indicate that physical inactivity acts predominantly during the reperfusion phase, amplifying the secondary inflammatory and apoptotic response rather than the initial ischemic insult. This distinction is relevant to prehabilitation strategies before liver surgery. Although cardiorespiratory fitness is an established predictor of postoperative outcomes across surgical populations [91], our experimental model suggests that merely being physically more active will not prevent the hepatocellular injury inherent to vascular clamping, but may attenuate the amplification that follows it and thus represents a modifiable determinant of hepatic resilience.

## Figures and Tables

**Table 1:** Effects of surgical condition (sham vs. ischemia) and physical activity (inactive vs. active) on markers of liver injury/function, apoptosis, inflammation, oxidative stress, endothelial dysfunction, mitochondrial biogenesis and dynamics, and systemic metabolic markers.

	SHAM				ISCHEMIA						
Variable	Physically Inactive (PI-S)	Physically Active (PA-S)			Physically Inactive (PI-IR)		Physically Active (PA-IR)	Intervention		PA	Interaction
	Mean ± SD	*n*	Mean ± SD	*n*	Mean ± SD	*n*	Mean ± SD	*n*	*p*	*p*	*p*
Liver injury/function											
Albumin (g/L)	3.90 ± 0.05 **^a^**	7	3.89 ± 0.09 **^a^**	7	3.79 ± 0.10	7	3.73 ± 0.07	7	**<0.001**	0.210	0.402
ALP (U/L)	6.89 ± 2.00	7	8.08 ± 2.64 **^a^**	7	11.0 ± 4.77 **^b^**	6	29.8 ± 17.9	7	**<0.001**	**0.003**	**0.021**
ALT (U/L)	115 ± 50.8 **^a^**	7	108 ± 56.8 **^a^**	7	576 ± 103	6	618 ± 490	7	**<0.001**	0.867	**NA**
Amylase (U/L)	732 ± 66.6	7	746 ± 70.7	7	745 ± 118	7	702 ± 99.5	7	0.663	0.682	0.425
AST (U/L)	245 ± 52.3 **^a^**	7	261 ± 99.8	7	511 ± 79.0	6	488 ± 263	7	**<0.001**	0.793	**NA**
Bile acids (μmol/L)	13.7 ± 6.95	7	14.3 ± 11.1 **^a^**	7	14.3 ± 5.69 **^b^**	6	33.2 ± 13.4	7	**0.018**	**0.018**	**0.026**
Direct bilirubin (mg/dL)	0.07 ± 0.01	7	0.07 ± 0.01	7	0.08 ± 0.01	6	0.10 ± 0.04	7	0.227	0.445	0.400
Total bilirubin (mg/dL)	0.05 ± 0.01 **^a^**	7	0.06 ± 0.01	7	0.07 ± 0.02	6	0.08 ± 0.02	7	**0.003**	0.532	0.685
**Apoptosis**											
Bax/Bcl-2 ratio (OD)	0.14 ± 0.04	6	0.12 ± 0.03 **^a^**	6	0.16 ± 0.04 **^b^**	6	0.08 ± 0.02	7	0.477	**0.004**	**0.026**
Bcl-2 (OD)	602 ± 196	6	456 ± 141	6	476 ± 70.5	6	706 ± 127	7	0.280	0.461	**0.003**
Bax (OD)	81.8 ± 32.8	6	56.1 ± 21.1	6	72.9 ± 14.3 **^b^**	6	57.8 ± 13.4	7	0.680	**0.028**	0.547
Calpain-2 (OD)	109 ± 14.1	6	103 ± 7.68	6	111 ± 11.5	6	104 ± 8.80	7	0.689	0.130	0.909
Caspase-3 (OD)	237 ± 133	6	237 ± 50.2	6	315 ± 111	6	224 ± 89.6	6	0.446	0.282	0.283
Caspase-8 (OD)	686 ± 81.1 **^b^**	6	504 ± 131	6	672 ± 80.2	6	549 ± 140	7	0.742	**0.003**	0.521
**Inflammation**											
CD68 (OD)	163 ± 8.70	6	169 ± 11.1	6	161 ± 16.0	6	158 ± 40.7	7	0.515	0.872	0.643
CD163 (OD)	287 ± 77.4	6	374 ± 153	6	284 ± 40.5	6	309 ± 101	7	0.414	0.183	0.454
iNOS (OD)	245 ± 163	6	161 ± 111	6	140 ± 71.4	6	174 ± 57.2	7	0.292	0.559	0.183
MPO activity (nmol·min^−1^·mL^−1^)	13.4 ± 2.60 **^a,b^**	7	4.26 ± 1.38 **^a^**	7	19.7 ± 5.88 **^b^**	7	9.76 ± 2.18	7	**<0.001**	**<0.001**	**0.035**
MPO (OD)	181 ± 181	6	62.3 ± 21.6	6	270 ± 334	6	92.1 ± 59.1	7	0.441	0.062	0.700
Nf-κB (OD)	429 ± 108	6	402 ± 113	6	419 ± 60.8	6	447 ± 218	7	0.767	0.998	0.637
TNF-α (OD)	232 ± 40.8 **^a^**	6	255 ± 84.6	6	349 ± 72.1 **^b^**	6	206 ± 75.7	7	0.243	**0.046**	**0.008**
**Oxidative stress**											
Catalase (OD)	84.7 ± 20.1	6	87.5 ± 16.1	6	88.1 ± 14.9	6	85.2 ± 18.6	7	0.936	0.991	0.695
Glutathione peroxidase 1 (OD)	86.6 ± 44.5	6	86.8 ± 34.6	6	72.6 ± 35.4	6	89.7 ± 26.6	7	0.701	0.550	0.557
3-NT (OD)	225 ± 51.8	6	186 ± 47.8	6	278 ± 130	6	207 ± 43.3	6	0.270	0.083	0.885
4-HNE (OD)	784 ± 173	6	672 ± 134	6	836 ± 169	6	693 ± 127	6	0.567	0.054	0.808
Protein carbonyls (OD)	331 ± 63.6	6	323 ± 86.8	6	387 ± 146	6	372 ± 103	6	0.230	0.783	0.930
SOD2 (OD)	171 ± 26.1	6	160 ± 9.27	6	153 ± 24.9	6	177 ± 27.6	7	0.968	0.494	0.076
**Endothelial dysfunction**											
Endothelin 1 (OD)	88.0 ± 33.6	6	92.8 ± 29.2	6	85.0 ± 22.2	6	99.3 ± 25.2	7	0.877	0.403	0.674
eNOS (OD)	104 ± 76.1	6	89.1 ± 24.5	6	100 ± 49.4	6	86.3 ± 27.7	7	0.865	0.457	0.979
ICAM1 (OD)	122 ± 23.2	6	116 ± 14.5	6	123 ± 18.7	6	113 ± 25.7	7	0.881	0.361	0.875
VCAM1 (OD)	223 ± 84.0	6	202 ± 77.0	6	161 ± 57.1	6	157 ± 57.3	7	0.065	0.667	0.758
**Mitochondrial biogenesis and dynamics**											
CS activity (nmol·min^−1^·mg^−1^)	53.5 ± 19.7	7	69.5 ± 16.1	7	51.5 ± 10.1**^b^**	7	75.0 ± 27.0	7	0.813	**0.012**	0.610
MFN1 (OD)	188 ± 65.2	6	154 ± 37.6	6	135 ± 34.3	6	190 ± 73.6	7	0.717	0.653	0.061
MFN2 (OD)	241 ± 86.1	6	207 ± 75.1	6	211 ± 69.6	6	289 ± 126	7	0.502	0.568	0.151
mtTFA (OD)	128 ± 48.3	6	107 ± 20.9	6	128 ± 42.3	6	139 ± 44.2	7	0.333	0.783	0.340
PGC1-α (OD)	202 ± 62.2	6	216 ± 42.0	6	183 ± 44.8	6	244 ± 83.7	7	0.867	0.148	0.354
**Systemic markers**											
Creatinine (mg·dL^−1^)	0.27 ± 0.03	7	0.26 ± 0.07	7	0.28 ± 0.05	7	0.29 ± 0.04	7	0.316	0.841	0.541
CK (U/L)	290 ± 180	7	166 ± 39.0	7	177 ± 52.4	7	196 ± 35.9	7	0.268	0.166	0.064
Glucose (mg·dL^−1^)	235 ± 32.1	7	237 ± 52.0	7	208 ± 38.5	7	203 ± 13.8	7	0.039	0.918	0.798
Lactate (mg·dL^−1^)	27.6 ± 6.22	7	26.5 ± 6.11	7	27.5 ± 5.80	7	29.9 ± 5.84	7	0.466	0.786	0.439

Values are presented as mean ± standard deviation, together with the number of animals per group (*n*), for each experimental condition: PI-S (physically inactive—sham), PA-S (physically active—sham), PI-IR (physically inactive ischemia–reperfusion) and PA-IR (physically active ischemia–reperfusion). Intervention, main effect of surgical condition; PA, main effect of physical activity; interaction, surgical procedure × activity interaction. **^a^** indicates statistically significant differences (*p* < 0.05) between sham and ischemia groups; **^b^** indicates statistically significant differences (*p* < 0.05) between physically inactive and physically active groups. Bold values indicate statistically significant differences (*p* < 0.05). NA, not applicable. ALP, alkaline phosphatase; ALT, alanine aminotransferase; AST, aspartate aminotransferase; Bax, Bcl-2 associated X protein; Bcl-2, B-cell lymphoma 2; iNOS, inducible nitric oxide synthase; MPO, myeloperoxidase; NF-κB, nuclear factor kappa B; TNF-α, tumor necrosis factor alpha; 3-NT, 3-nitrotyrosine; 4-HNE, 4-hydroxynonenal; SOD2, superoxide dismutase 2 (manganese superoxide dismutase); eNOS, endothelial nitric oxide synthase; ICAM1, intercellular adhesion molecule 1; VCAM1, vascular cell adhesion molecule 1; CS, citrate synthase; MFN1, mitofusin 1; MFN2, mitofusin 2; mtTFA, mitochondrial transcription factor A; PGC1-α, peroxisome proliferator-activated receptor gamma coactivator 1-alpha; CK, creatine kinase; OD, optical density; U/L, units per liter.

## Data Availability

The original contributions presented in this study are included in the article/Supplementary Materials. Further inquiries can be directed to the corresponding authors.

## Supplementary Materials

The following supporting information can be downloaded at https://www.mdpi.com/article/10.3390/biology15201850/s1: Figure S1. Representative slot-blot membranes for protein carbonylation (DNP), 3-nitrotyrosine (3-NT), and 4-hydroxynonenal (4-HNE), followed by Ponceau S loading controls and uncropped Western blot membranes for mtTFA, MFN2, MFN1, PGC1-α, Bcl-2, Bax, Calpain-2, Caspase-8, Caspase-3, MPO, TNF-α, NF-κB, GPx1, SOD2, VCAM-1, eNOS, CD68, CD163, Catalase, Endothelin-1, ICAM-1 and iNOS; Table S1. O2k manual titrations of the SUIT-009 AmR mt D021 protocol for liver tissue homogenates in the O2k-sVModule-Mitochondrial Physiology Network (Oroboros Instruments, Innsbruck, Austria); Table S2. Differences in food intake between groups (Šídák’s test); Table S3. Individual running wheel distance of each physically active animal throughout the pre-surgical intervention period. Values represent the mean daily running distance (meters/day) recorded in each week for each animal of the PA-S (*n* = 7) and PA-IR (*n* = 7) groups.

## Abbreviations

The following abbreviations are used in this manuscript:

3-NT	3-nitrotyrosine
4-HNE	4-hydroxynonenal
ADP	adenosine diphosphate
ALP	alkaline phosphatase
ALT	alanine aminotransferase
AmR	Amplex UltraRed
ANOVA	analysis of variance
AST	aspartate aminotransferase
ATP	adenosine triphosphate
Bax	Bcl-2 associated X protein
Bcl-2	B-cell lymphoma 2
BSA	bovine serum albumin
CD68	cluster of differentiation 68
CD163	cluster of differentiation 163
CK	creatine kinase
CS	citrate synthase
DAMPs	damage-associated molecular patterns
DNP	2,4-dinitrophenyl
DNPH	2,4-dinitrophenylhydrazine
DTPA	diethylenetriaminepentaacetic acid
DTNB	5,5′-dithiobis-(2-nitrobenzoic acid)
ECL	enhanced chemiluminescence
eNOS	endothelial nitric oxide synthase
ET-1	endothelin-1
FELASA	Federation of European Laboratory Animal Science Associations
HIIT	high-intensity interval training
HRP	horseradish peroxidase
H_2_O_2_	hydrogen peroxide
ICAM1	intercellular adhesion molecule 1
IgG	immunoglobulin G
iNOS	inducible nitric oxide synthase
IP	intraperitoneal
IR	ischemia–reperfusion
IRI	ischemia–reperfusion injury
JO_2_	oxygen flux
MFN1	mitofusin 1
MFN2	mitofusin 2
MPO	myeloperoxidase
mtTFA	mitochondrial transcription factor A
NF-κB	nuclear factor kappa B
NS-pathway	NADH-linked and succinate-linked pathway
OD	optical density
OXPHOS	oxidative phosphorylation
PA-IR	physically active ischemia–reperfusion (group)
PA-S	physically active—sham (group)
PGC1-α	peroxisome proliferator-activated receptor gamma coactivator 1-alpha
PI-IR	physically inactive-ischemia–reperfusion (group)
PI-S	physically inactive—sham (group)
RCR	respiratory control ratio
RET	reverse electron transport
ROS	reactive oxygen species
ROUT	robust regression and outlier removal
ROX	residual oxygen consumption
SDS-PAGE	sodium dodecyl sulfate-polyacrylamide gel electrophoresis
SOD	superoxide dismutase
SOD2	superoxide dismutase 2 (manganese superoxide dismutase)
SUIT	substrate-uncoupler-inhibitor titration (protocol)
TBS	Tris-buffered saline
TBS-T	Tris-buffered saline with Tween-20
TCA	tricarboxylic acid cycle
TNB	2-nitro-5-thiobenzoic acid
TNF-α	tumor necrosis factor alpha
TUNEL	terminal deoxynucleotidyl transferase dUTP nick end labeling
VCAM1	vascular cell adhesion molecule 1
