# Supplementary material for: Does Physical Inactivity Aggravate Liver Ischemia–Reperfusion Injury? Evidence from a Preclinical Wistar Rat Model
Source: Biology (Basel). 2026 Oct 15;15(20):1850. doi: 10.3390/biology15201850 (PMC13701282; doi:10.3390/biology15201850)
Supplement: Supplementary file 1 [file biology-15-01850-s001.zip › Figure_S1_Manuscript_Biology_IRI_Miguel_Anjos.pdf]

Figure S1

Article

Does physical inactivity aggravate liver ischemia-reperfusion injury? Evidence from a preclinical Wistar rat model

Miguel Anjos[1,2,*], Pedro Silva Oliveira[1,2,3], Diogo Duarte Carvalho[1,2], Ariane Flores[1,2], Sophia Ramos[4], Rita Ferreira[5], Ana Isabel Padrão[1,2], Ana Reis-Mendes[1,2,*] and Hélder Fonseca[1,2]

Figure S1. Representative slot blot membranes for protein carbonylation (DNP), 3-nitrotyrosine (3-NT), and 4-hydroxynonenal (4-HNE), followed by Ponceau S loading controls and uncropped western blot membranes for mtTFA, MFN2, MFN1, PGC1-α, Bcl-2, Bax, Calpain-2, Caspase-8, Caspase-3, MPO, TNF-α, NF-κB, GPx1, SOD2, VCAM-1, eNOS, CD68, CD163, Catalase, Endothelin-1, ICAM-1 and iNOS.

Figure S2

Uncropped slot blot for DNP (MAB2223)

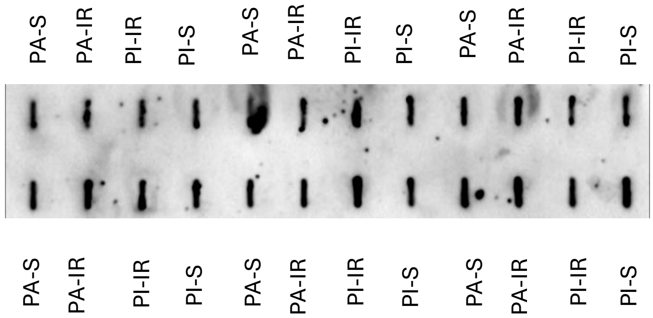

Ponceau S

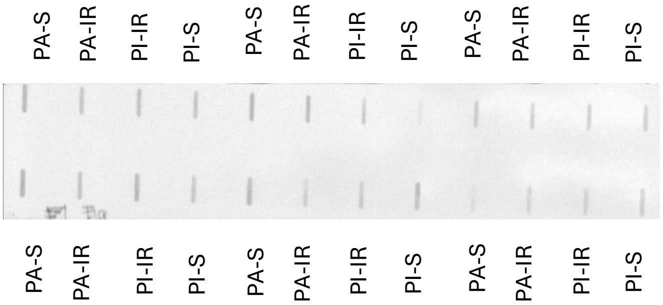

Uncropped slot blot for 3-nitrotyrosine (MAB5404)

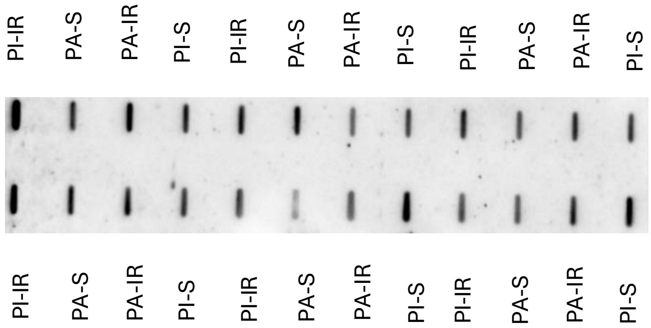

Ponceau S

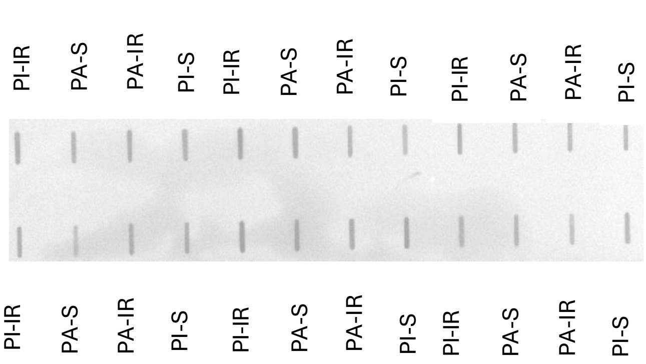

Uncropped slot blot
for 4-hydroxynonenal (ab46545)

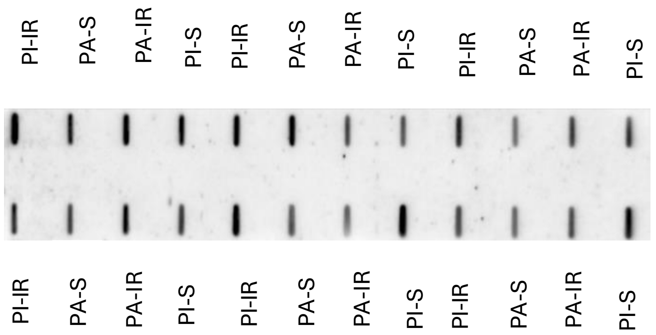

Ponceau S

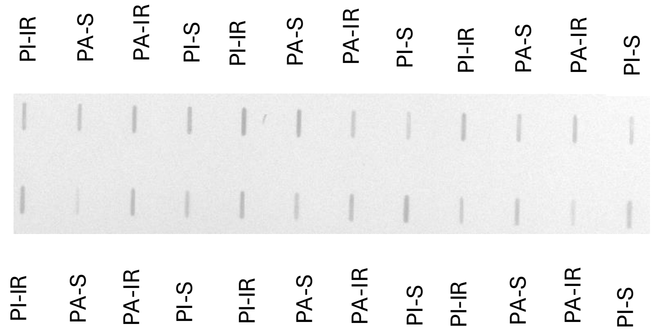

Western blot for mtTFA (sc-376672)
Specific band: 25 kDa

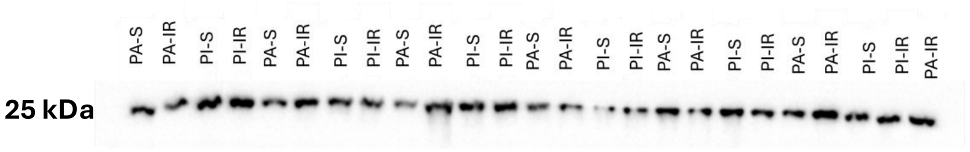

Ponceau S

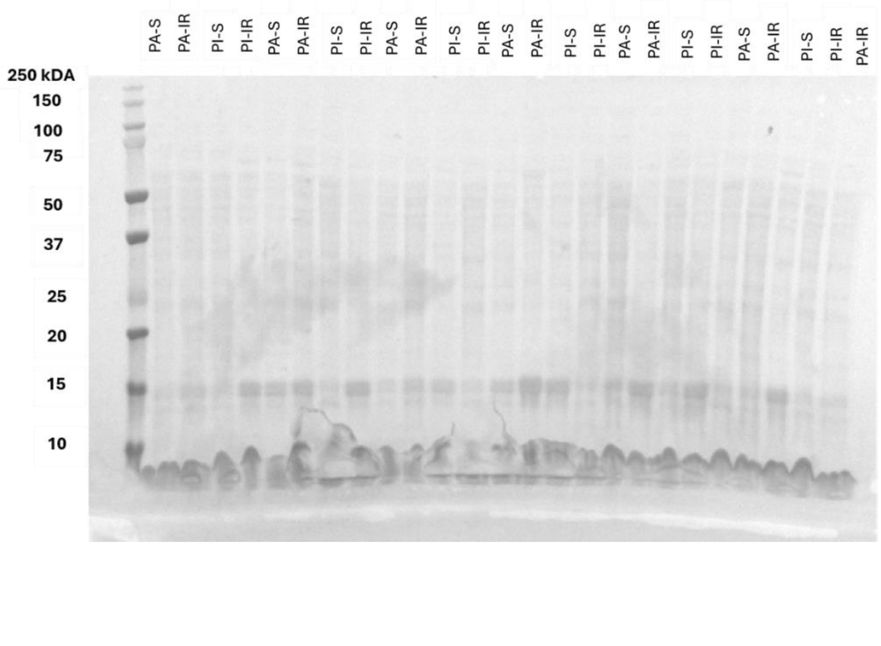

Western blot for MFN2 (sc-515647)
Specific band: 86 kDa

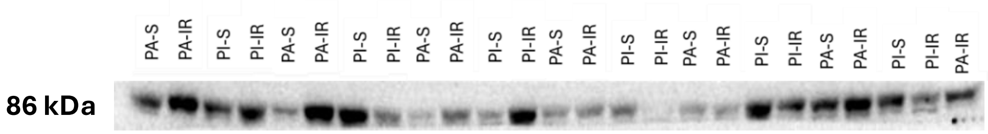

Ponceau S

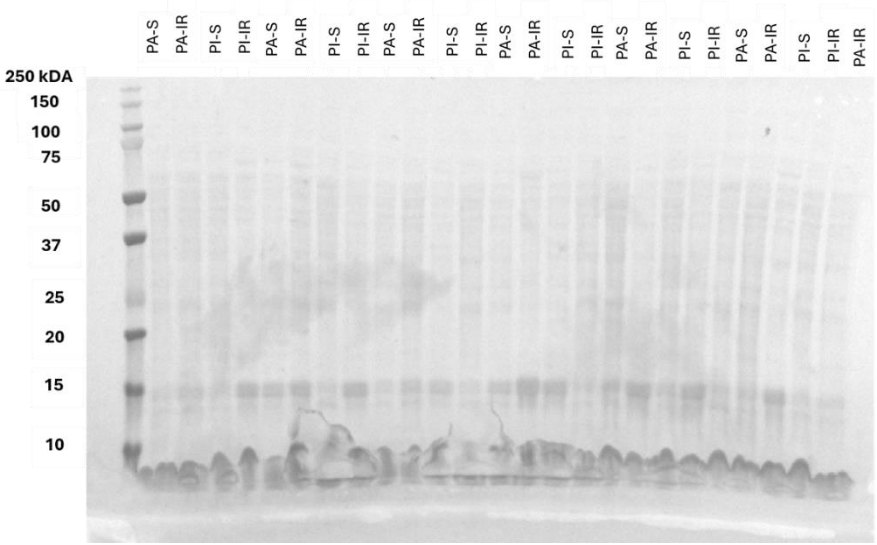

Western blot for MFN1 (sc-166644)
Specific band: 86 kDa

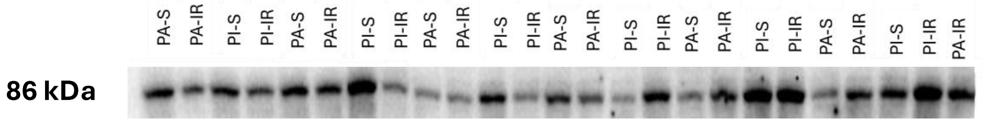

Ponceau S

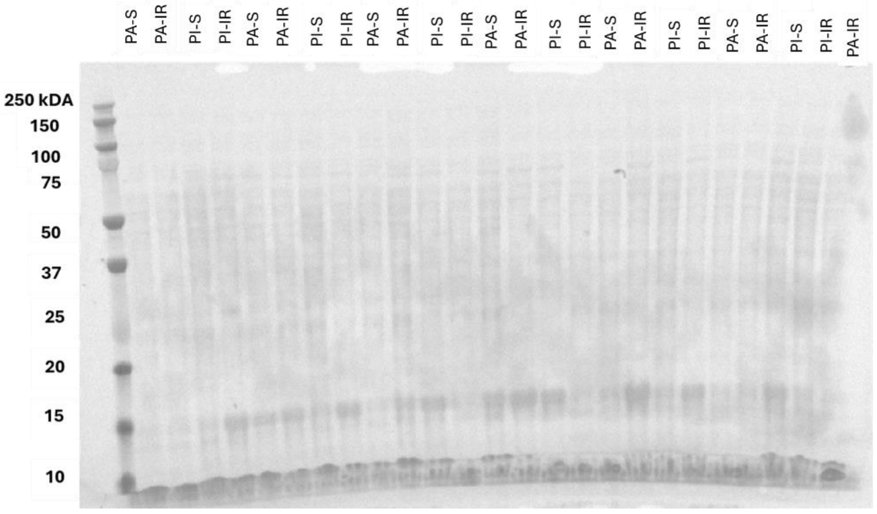

Western blot for PGC1-α (ab54481)
Specific band: 92 kDa

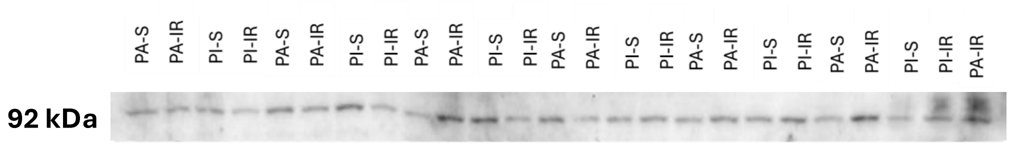

Ponceau S

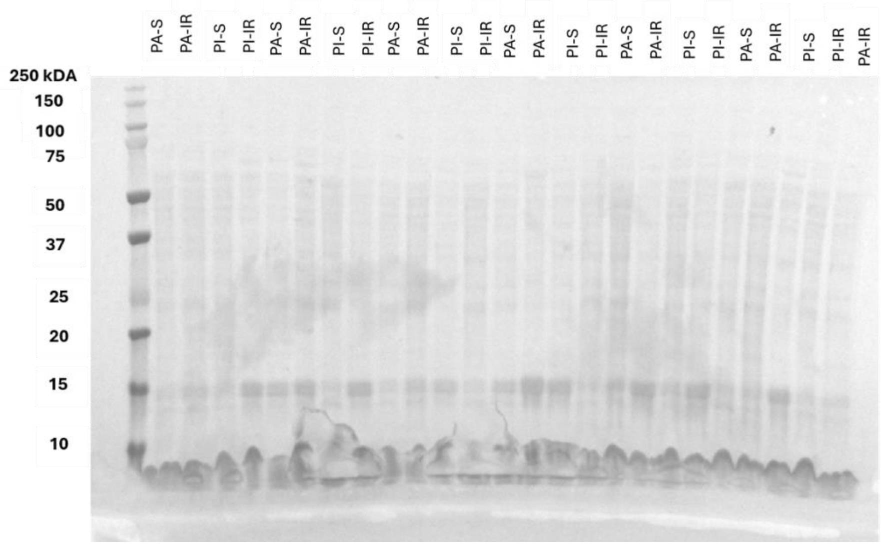

Western blot for Bcl-2 (ab16904)
Specific band: 26 kDa

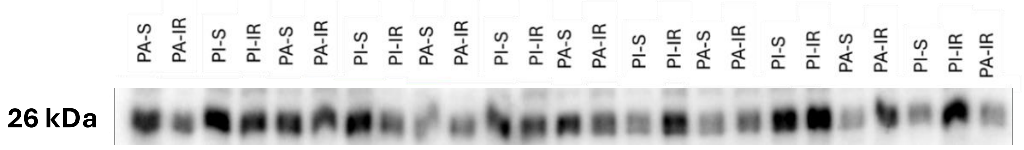

Ponceau S

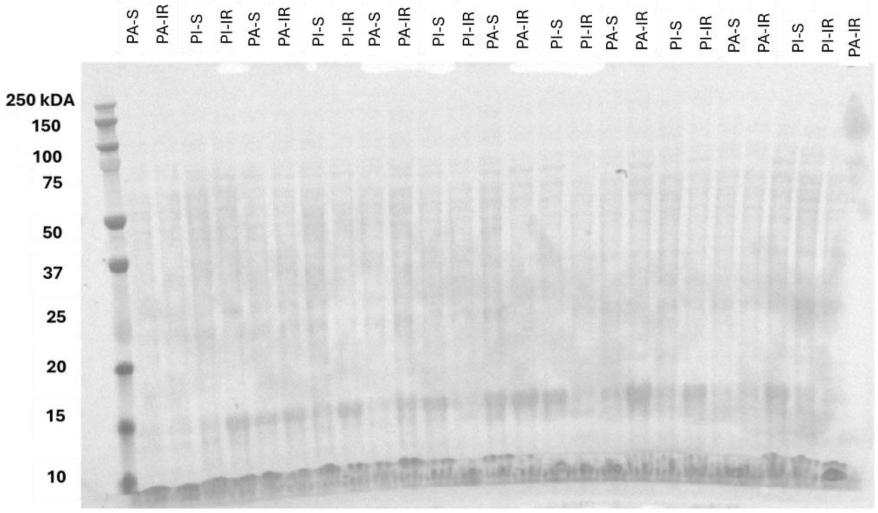

Western blot for Bax (ab32503)
Specific band: 21 kDa

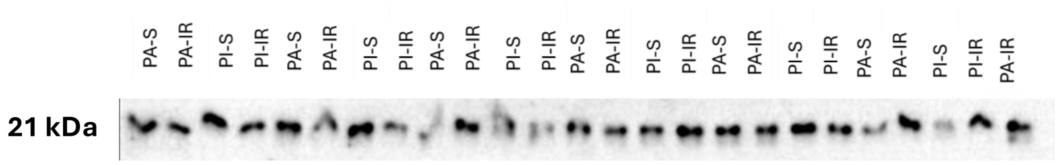

Ponceau S

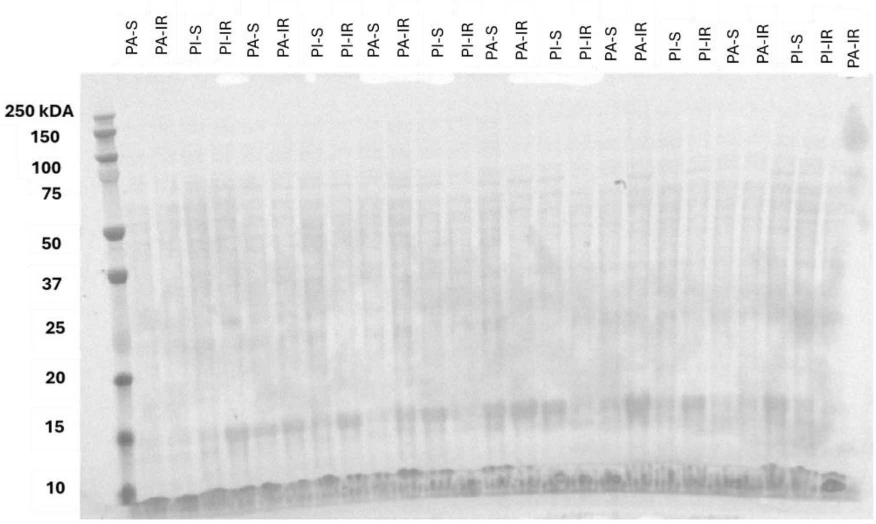

Western blot for Calpain-2 (ab39165)
Specific band: 80 kDa

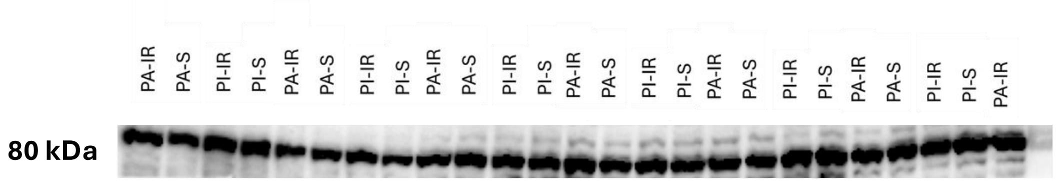

Ponceau S

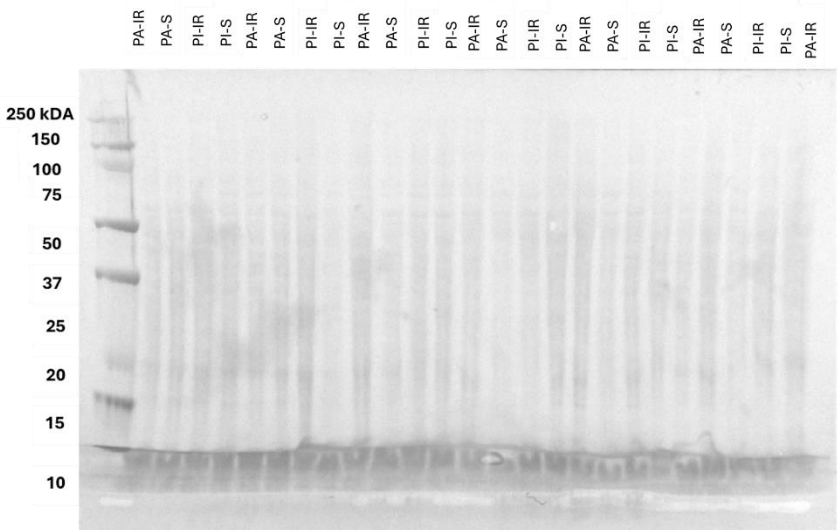

Western blot for Caspase-8 (ab25901)
Specific band: 55 kDa

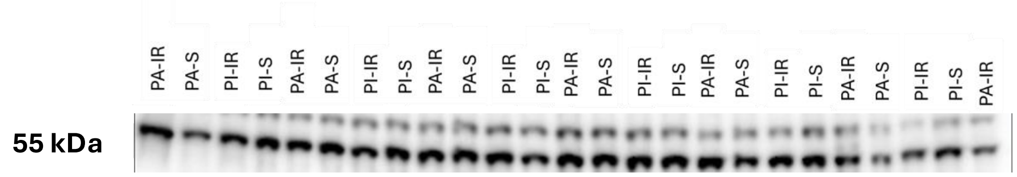

Ponceau S

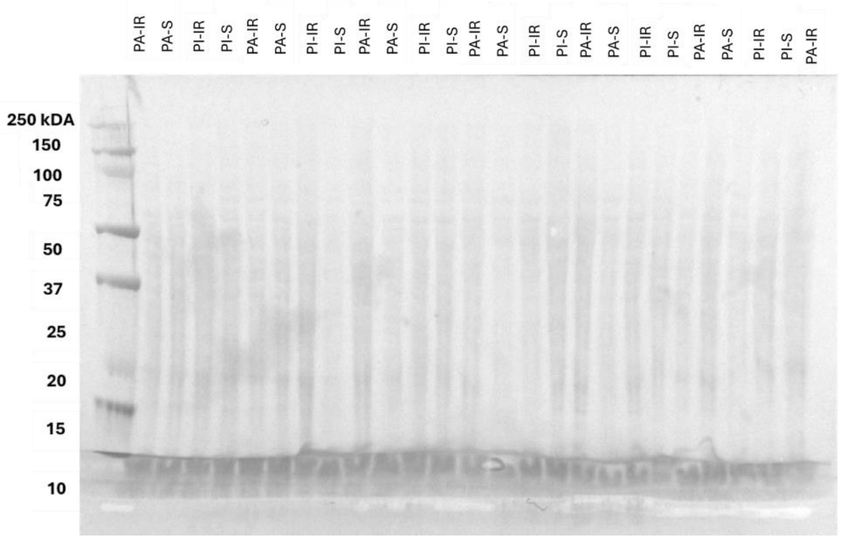

Western blot for Caspase-3 (ab13847)
Specific band: 31 kDa

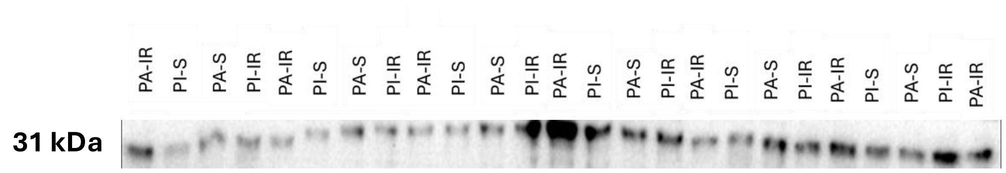

Ponceau S

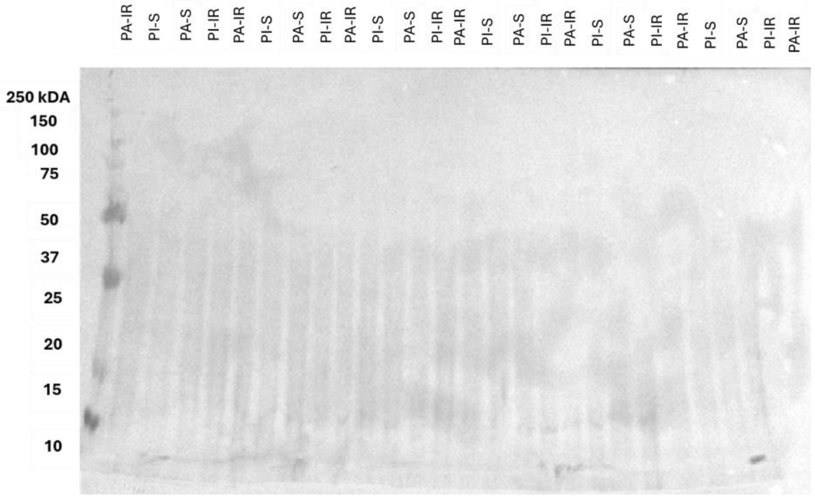

Western blot for MPO (ab208670)
Specific band: 59 kDa

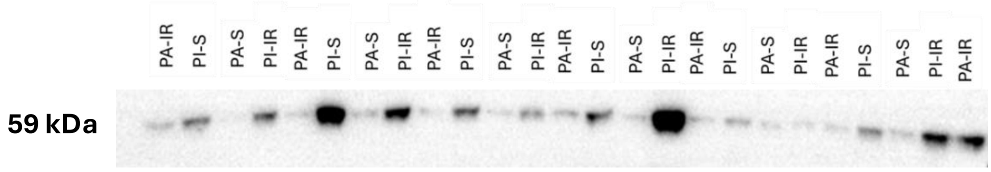

Ponceau S

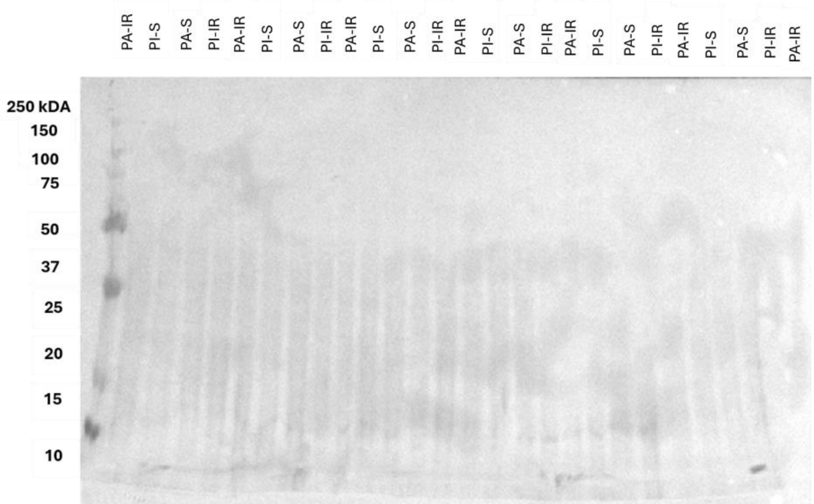

Western blot for TNF-α (ab6671)
Specific band: 25 kDa

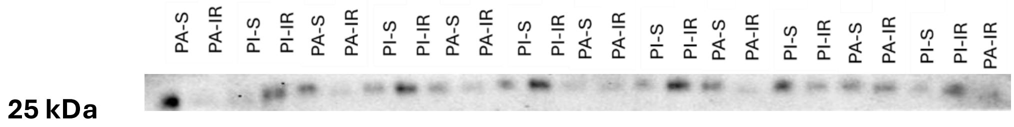

Ponceau S

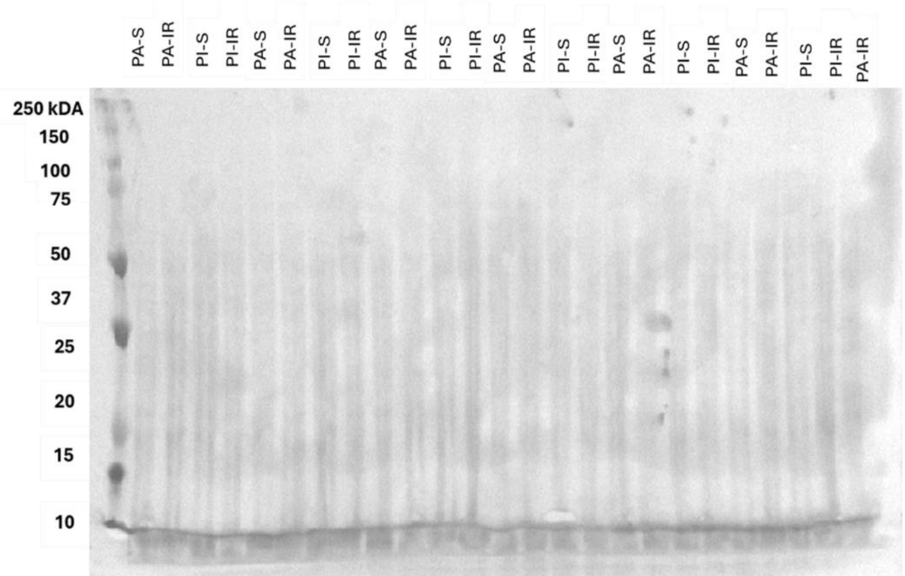

Western-blot for NF-kB (sc-114)
Specific band: 50 kDa

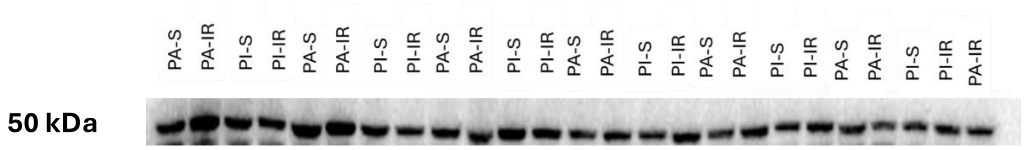

Ponceau S

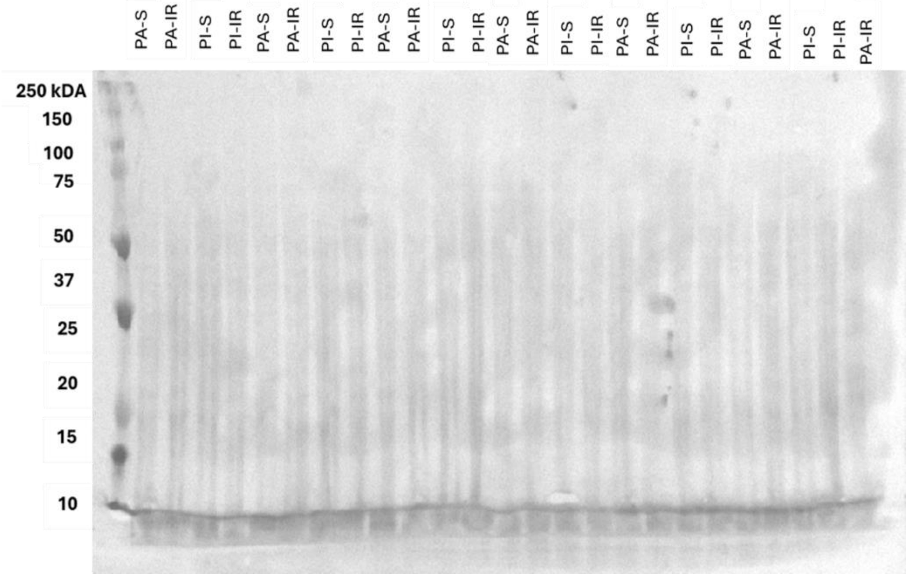

Western blot for Glutathione peroxidase 1 (ab22604)
Specific band: 55 kDa

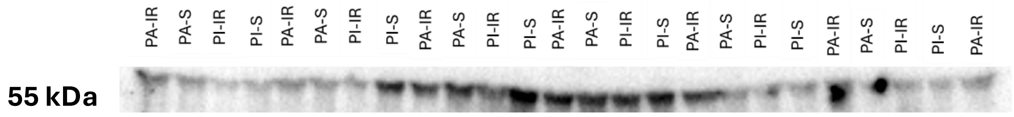

Ponceau S

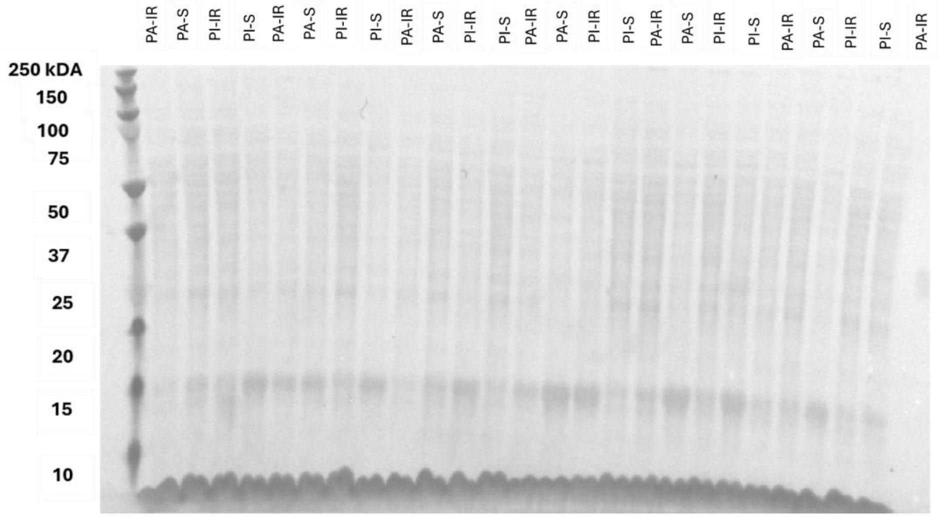

Western blot for SOD2 (ab13534)
Specific band: 24 kDa

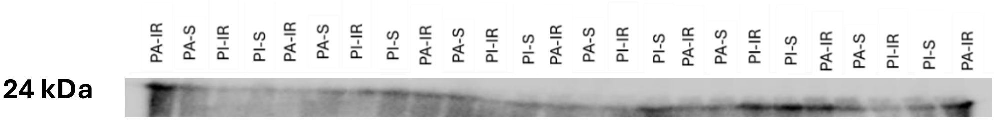

Ponceau S

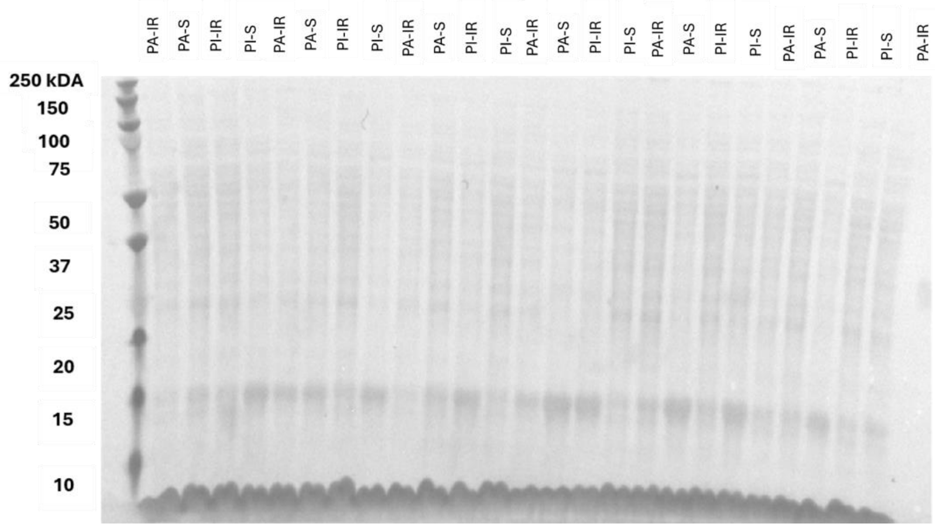

Western blot for VCAM1 (ab134047)
Specific band: 81 kDa

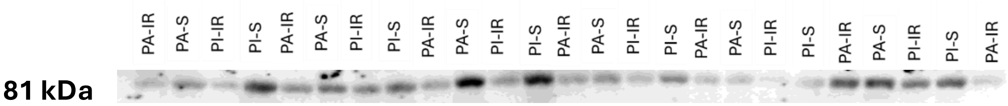

Ponceau S

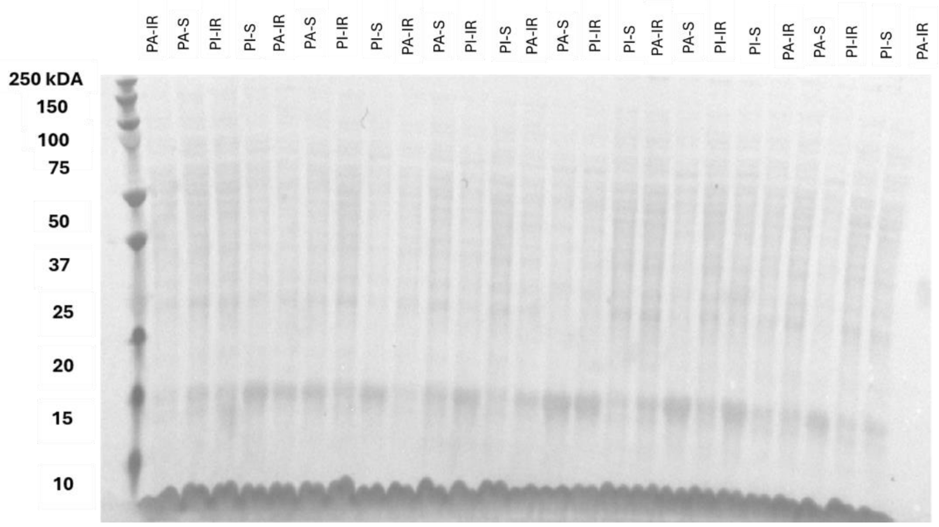

Western blot for eNOS (ab199956)
Specific band: 133 kDa

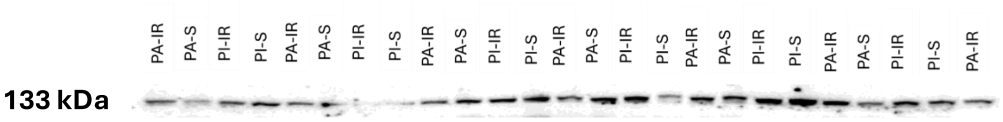

Ponceau S

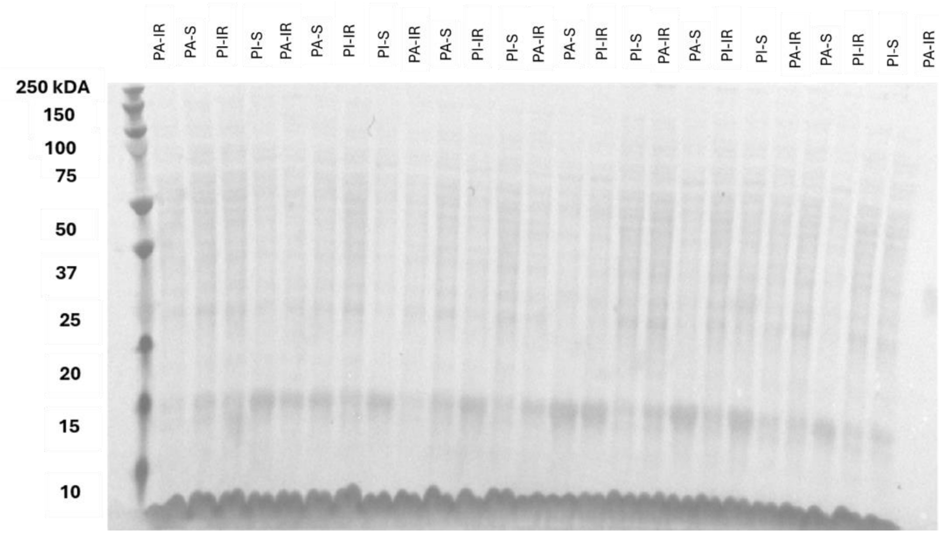

Western blot for CD68 (ab125212)
Specific band: 100 kDa

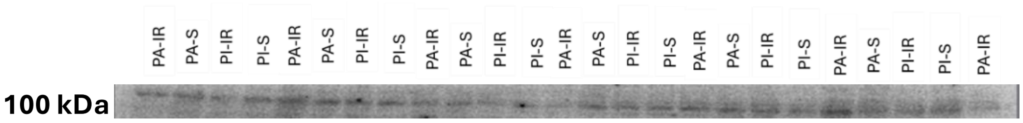

Ponceau S

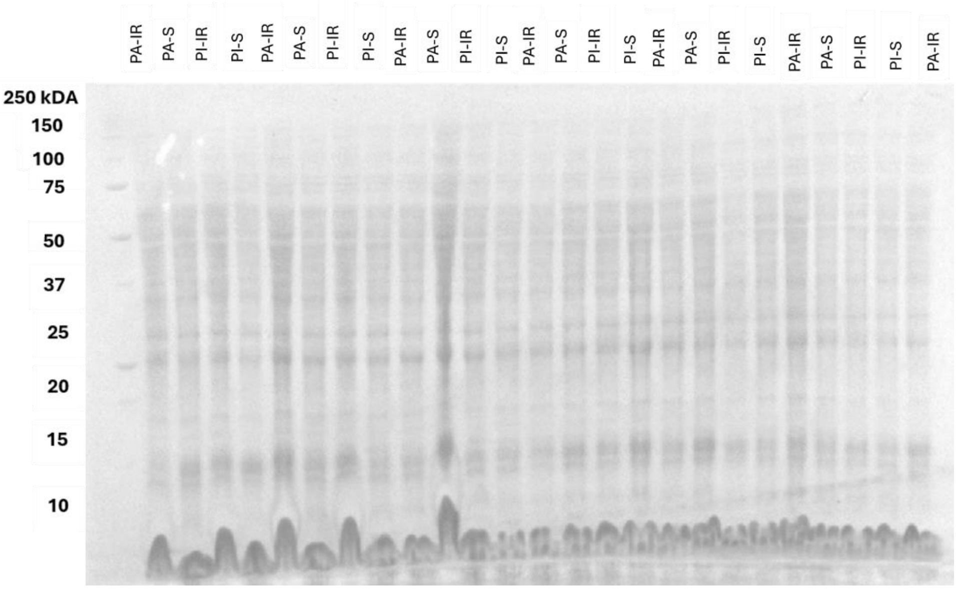

Western blot for CD163 (ab182422)
Specific band: 125 kDa

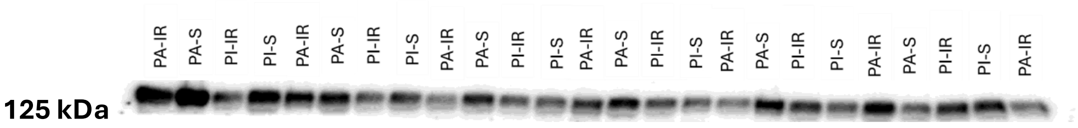

Ponceau S

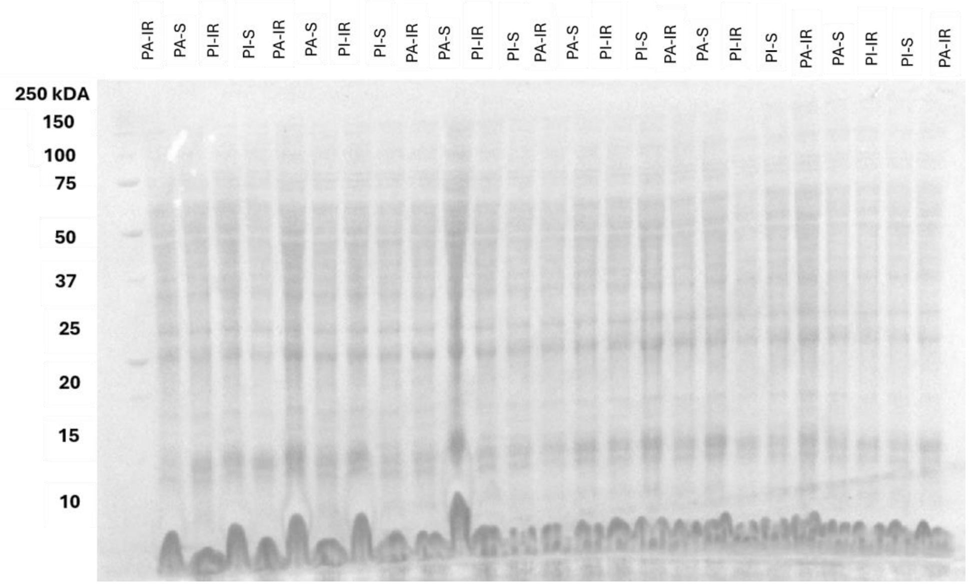

Western blot for Catalase (ab16731)
Specific band: 60 kDa

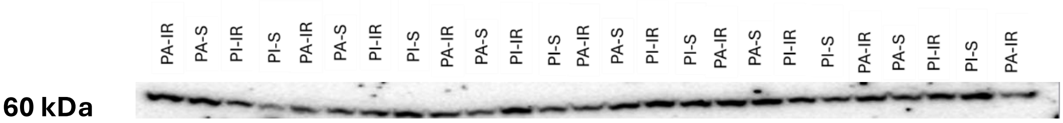

Ponceau S

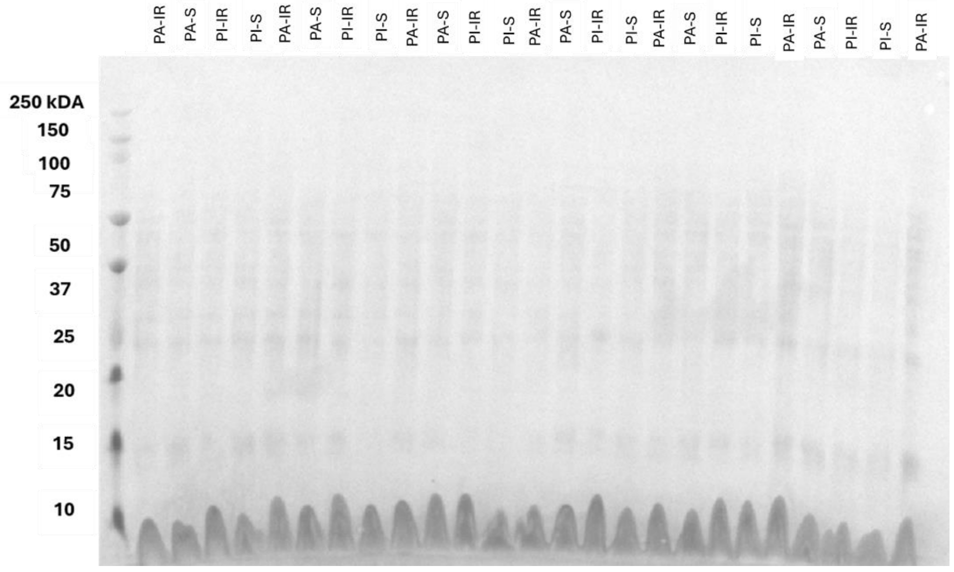

Western blot for Endothelin 1 (ab2786)
Specific band: 24 kDa

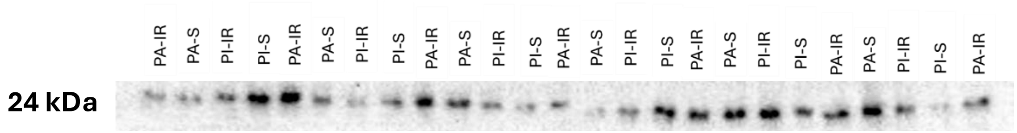

Ponceau S

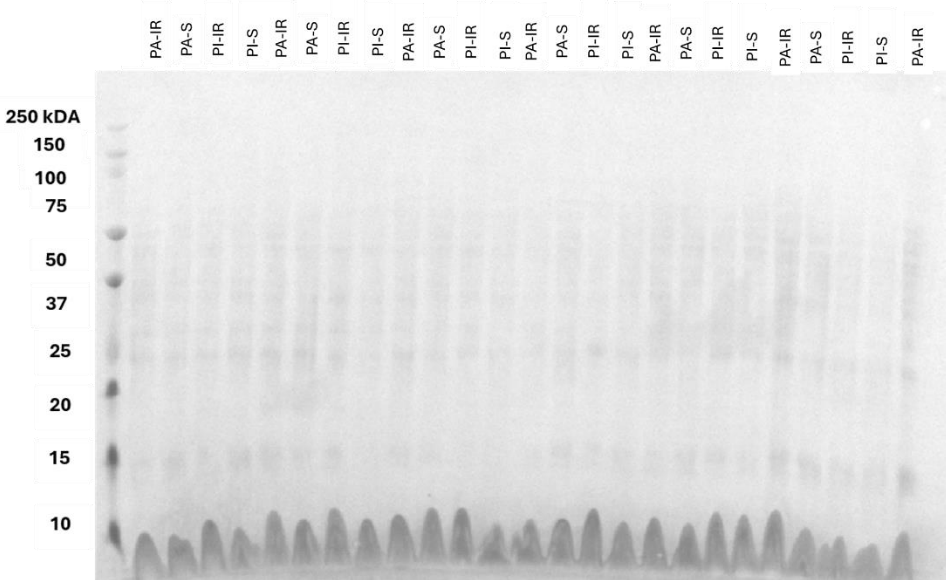

Western blot for ICAM1 (ab171123)
Specific band: 57 kDa

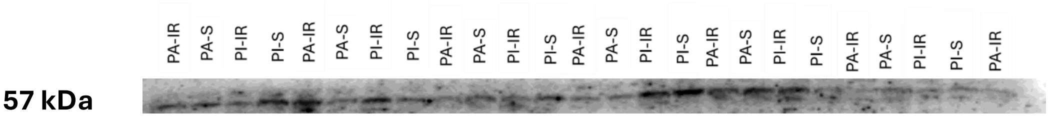

Ponceau S

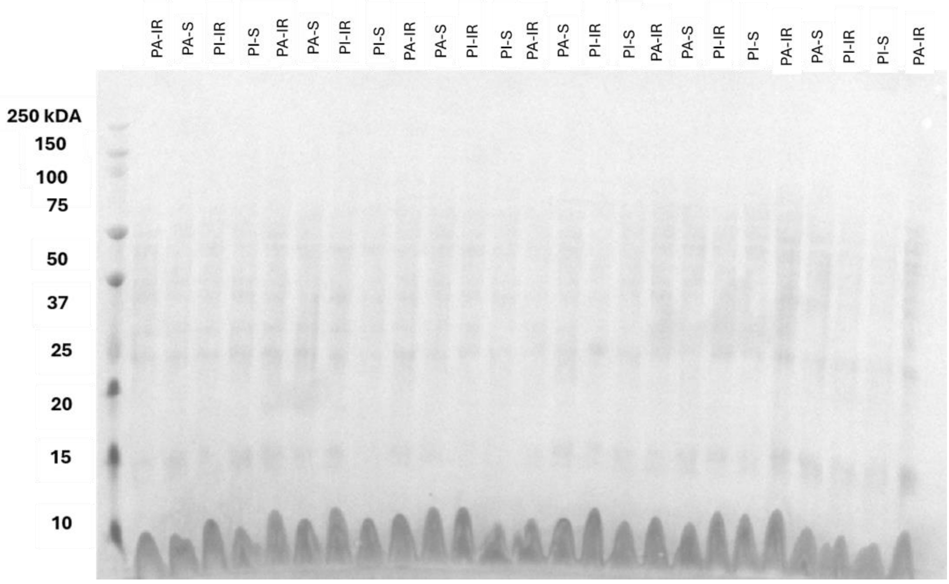

Western blot for iNOS (ab283655)
Specific band: 131 kDa

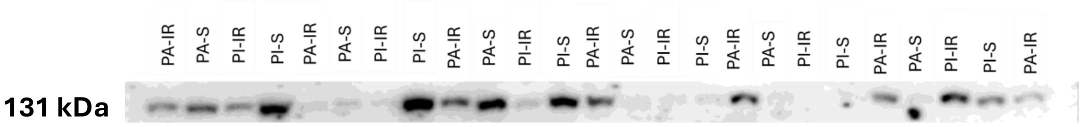

Ponceau S

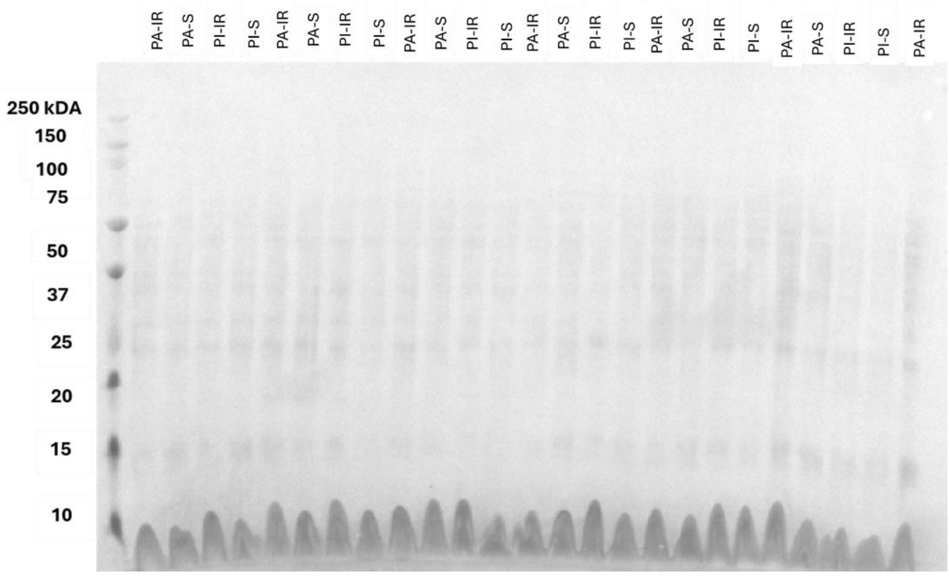

Legend: Membranes were cut horizontally according to the molecular weight of each target before antibody incubation. Therefore, targets detected on the same membrane share the same Ponceau S image. PI-S, physically inactive-sham; PA-S, physically active-sham; PI-IR, physically inactive-ischemia-reperfusion; PA-IR, physically active-ischemia-reperfusion.
