# Supplementary material for: Does Physical Inactivity Aggravate Liver Ischemia–Reperfusion Injury? Evidence from a Preclinical Wistar Rat Model
Source: Biology (Basel). 2026 Oct 15;15(20):1850. doi: 10.3390/biology15201850 (PMC13701282; doi:10.3390/biology15201850)
Supplement: Supplementary file 1 [file biology-15-01850-s001.zip › Table_S1_Manuscript Biology_IRI_Miguel Anjos.pdf]

Table S1

Article

Does physical inactivity aggravate liver ischemia-reperfusion injury? Evidence from a preclinical Wistar rat model

Miguel Anjos[1,2,*], Pedro Silva Oliveira[1,2,3], Diogo Duarte Carvalho[1,2], Ariane Flores[1,2], Sophia Ramos[4], Rita Ferreira[5], Ana Isabel Padrão[1,2], Ana Reis-Mendes[1,2,*] and Hélder Fonseca[1,2]

Table S1 - O2k manual titrations of the SUIT-009 AmR mt D021 protocol for liver tissue homogenates in the O2k-sVModule – Mitochondrial Physiology Network (Oroboros Instruments, Innsbruck, Austria).

Substrates	Event	Concentration (solvent)	Storage [°C]	Final concentration in 2 mL	Titration [µL]	Syringe [µL]
DTPA	DTPA	5mM (H_2O)	-20	5mM	6	10
Horseradish peroxidase	HRP	500U/mL (MiR05)	-20	1U/mL	4	10
Superoxide dismutase	SOD	5000U/mL (H_2O)	-20	5U/mL	2	10
Amplex UltraRed	AmR	10mM (DMSO)	-20	50µM	5	10
Hydrogen Peroxide	H_2O_2	880mM (HCL)	-20	40µM	5	10
Succinate	S	1M (H_2O)	-20	10mM	20	50
ADP + Mg^{2+}	D	500mM (H_2O)	-20	2.5mM	10	25
Pyruvate	P	2M (H_2O)	Fresh	5mM	5	25
Rotenone	Rot	1mM (EtOH)	-20	0.5µM	1	10
Antimycin A	Ama	5mM (EtOH)	-20	2.5mM	1	10

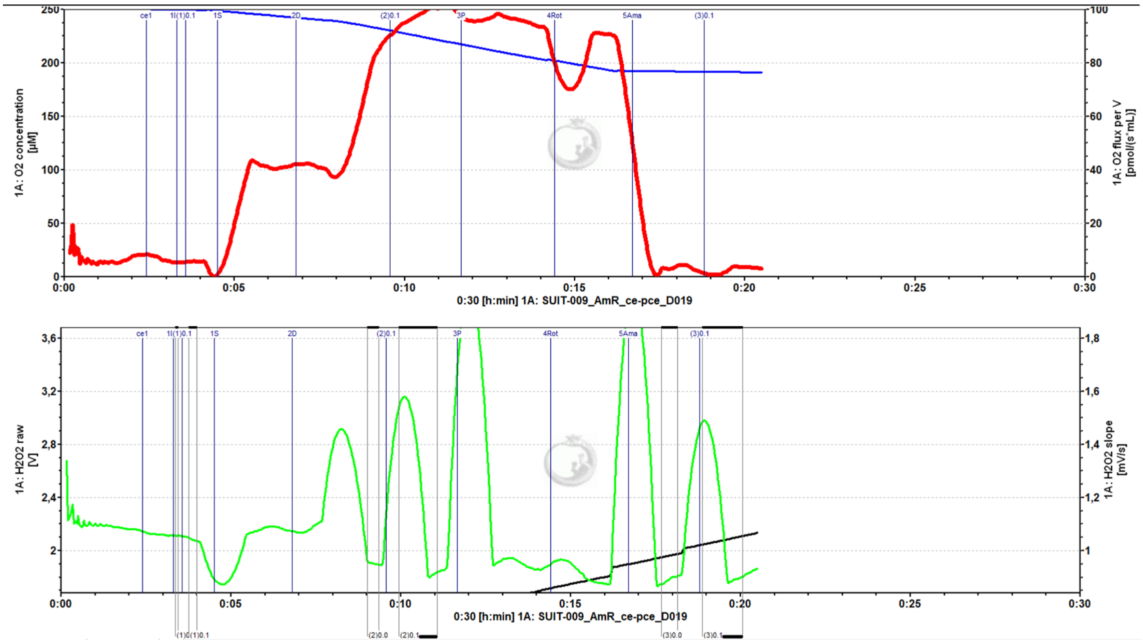

Note: Ama, Antimycin A (complex III inhibitor); AmR, Amplex UltraRed; D, Adenosine diphosphate (ADP) + Mg^{2+}; DMSO, Dimethyl sulfoxide; DTPA, Diethylenetriaminepentaacetic acid; EtOH, Ethanol; H_2O, Water; H_2O_2, Hydrogen peroxide; HRP, Horseradish peroxidase; P, Pyruvate (fresh preparation); Rot, Rotenone (complex I inhibitor); S, Succinate; SOD, Superoxide dismutase.
