# Supplementary material for: Does Physical Inactivity Aggravate Liver Ischemia–Reperfusion Injury? Evidence from a Preclinical Wistar Rat Model
Source: Biology (Basel). 2026 Oct 15;15(20):1850. doi: 10.3390/biology15201850 (PMC13701282; doi:10.3390/biology15201850)
Supplement: Supplementary file 1 [file biology-15-01850-s001.zip › Table_S2_Manuscript Biology_IRI_Miguel Anjos.pdf]

Table S2

Article

Does physical inactivity aggravate liver ischemia-reperfusion injury? Evidence from a preclinical Wistar rat model

Miguel Anjos[1,2,*], Pedro Silva Oliveira[1,2,3], Diogo Duarte Carvalho[1,2], Ariane Flores[1,2], Sophia Ramos[4], Rita Ferreira[5], Ana Isabel Padrão[1,2] , Ana Reis-Mendes[1,2,*] and Hélder Fonseca[1,2]

Table S2. Differences in food intake between groups (Šídák's test).

Week	PA-S vs. PI-S	PA-S vs. PI-IR	PA-IR vs. PI-S	PA-IR vs. PI-IR	PA-S vs. PA-IR	PI-S vs. PI-IR
**1**	n.s.	n.s.	n.s.	n.s.	n.s.	n.s.
**3**	****	n.s.	***	n.s.	n.s.	n.s.
**5**	****	****	****	*	n.s.	n.s.
**7**	****	****	***	*	n.s.	n.s.
**9**	****	***	***	*	n.s.	n.s.
**11**	****	**	**	*	n.s.	n.s.
**13**	****	***	*	n.s.	n.s.	n.s.
**15**	****	**	n.s.	n.s.	n.s.	n.s.
**17**	***	*	*	n.s.	n.s.	n.s.
**19**	**	n.s.	**	n.s.	n.s.	n.s.
**21**	**	**	n.s.	n.s.	n.s.	n.s.
**23**	*	**	n.s.	n.s.	n.s.	n.s.
**25**	n.s.	n.s.	n.s.	n.s.	n.s.	n.s.

Two-way ANOVA with Šídák's post-test (n = 7/group). Only significant comparisons shown; n.s. = not significant. *$p < 0.05$; **$p < 0.01$; ***$p < 0.001$; ****$p < 0.0001$. PI-S (physically inactive-sham), PA-S (physically active-sham), PI-IR (physically inactive-ischemia) and PA-IR (physically active-ischemia).
