# Supplementary material for: Does Physical Inactivity Aggravate Liver Ischemia–Reperfusion Injury? Evidence from a Preclinical Wistar Rat Model
Source: Biology (Basel). 2026 Oct 15;15(20):1850. doi: 10.3390/biology15201850 (PMC13701282; doi:10.3390/biology15201850)
Supplement: Supplementary file 1 [file biology-15-01850-s001.zip › Table_S3_Manuscript_Biology_IRI_Miguel_Anjos.pdf]

Table S3

Article

Does physical inactivity aggravate liver ischemia-reperfusion injury? Evidence from a preclinical Wistar rat model

Miguel Anjos[1,2,*], Pedro Silva Oliveira[1,2,3], Diogo Duarte Carvalho[1,2], Ariane Flores[1,2], Sophia Ramos[4], Rita Ferreira[5], Ana Isabel Padrão[1,2], Ana Reis-Mendes[1,2,*] and Hélder Fonseca[1,2]

Table S3. Individual running wheel distance of each physically active animal throughout the pre-surgical intervention period (mean daily distance per week, meters/day).

**PA-S (n = 7)**								
**Week**	**Rat 1**	**Rat 2**	**Rat 3**	**Rat 4**	**Rat 5**	**Rat 6**	**Rat 7**	**Mean ± SD**
**1**	1175	2218	1161	1384	1558	2465	1119	1583 ± 545
**2**	3240	3055	1758	1711	2192	2931	2074	2423 ± 639
**3**	4825	5201	2348	2834	3158	5166	2208	3677 ± 1339
**4**	5831	3951	2102	2121	3163	3901	2264	3333 ± 1360
**5**	6377	5207	2756	3266	3622	5099	3370	4242 ± 1325
**6**	5887	4773	1548	2950	3614	4468	3126	3766 ± 1416
**7**	4865	6624	1717	3702	4782	3899	3973	4223 ± 1484
**8**	4860	5602	1777	3492	4323	3592	4219	3981 ± 1213
**9**	6317	5979	2128	3483	4369	4519	3892	4384 ± 1440
**10**	3657	2125	1154	1787	2264	1878	1737	2086 ± 777
**11**	5454	2635	930	2140	2932	2378	2667	2734 ± 1365
**12**	5752	2285	849	1979	2767	1867	1665	2452 ± 1569
**13**	5239	2012	610	1753	2988	1665	2118	2341 ± 1459
**14**	5094	1741	571	1696	1899	1299	1190	1927 ± 1466
**15**	3163	1230	882	1631	2117	1301	1166	1641 ± 778
**16**	2814	1674	513	1469	1951	1533	1028	1569 ± 722
**17**	2678	1731	586	1648	2136	1387	967	1590 ± 701
**18**	2030	1716	512	1083	2237	1437	923	1420 ± 621
**19**	1823	1174	374	730	1740	1288	882	1144 ± 528
**20**	1919	1249	397	1182	1340	1193	809	1156 ± 470
**21**	1668	1031	350	1115	1245	866	778	1008 ± 410
**22**	1880	703	341	978	1363	967	685	988 ± 505
**23**	1753	784	301	1026	1348	843	695	964 ± 471
**24**	1406	696	291	925	1313	619	558	830 ± 408
**25**	1559	545	337	863	929	652	575	780 ± 397
**1–25**	**3651**	**2638**	**1052**	**1878**	**2454**	**2289**	**1788**	**2250 ± 811**

PA-IR (n = 7)								
**Week**	**Rat 1**	**Rat 2**	**Rat 3**	**Rat 4**	**Rat 5**	**Rat 6**	**Rat 7**	**Mean ± SD**
**1**	2737	898	971	838	1570	1203	1759	1425 ± 675
**2**	1837	1469	1457	1173	1909	2117	3953	1988 ± 924
**3**	2232	2005	2466	1456	2551	2425	6093	2747 ± 1522
**4**	2529	2132	2149	1430	1972	1996	8409	2945 ± 2431
**5**	3285	2767	2340	1466	2498	2448	11218	3717 ± 3352
**6**	3221	2685	2375	1383	2384	2549	9460	3437 ± 2712
**7**	3833	3648	2461	1319	2979	2749	9875	3838 ± 2788
**8**	3404	3591	2785	1128	1644	2419	7201	3167 ± 1987
**9**	2270	2597	2522	1290	1759	2381	9845	3238 ± 2950
**10**	1581	1629	1317	615	777	1289	5466	1811 ± 1656
**11**	1626	1837	1865	705	916	1519	6114	2083 ± 1832
**12**	1752	1328	1789	747	753	1250	6857	2068 ± 2153
**13**	2014	1689	1611	552	854	1384	6412	2074 ± 1977
**14**	1950	1094	1347	489	564	1303	6129	1840 ± 1956
**15**	2517	1207	1869	496	673	953	6677	2056 ± 2156
**16**	1092	1075	1529	463	593	1069	7252	1868 ± 2400
**17**	1614	1171	1642	478	587	972	6466	1847 ± 2086
**18**	1042	1202	1380	504	454	1114	5294	1570 ± 1679
**19**	1004	1040	1268	389	405	857	3854	1259 ± 1190
**20**	989	1019	1160	410	424	829	5364	1456 ± 1747
**21**	719	817	923	388	404	632	5232	1302 ± 1745
**22**	775	778	715	387	389	672	5847	1366 ± 1983
**23**	834	801	788	385	316	535	5974	1376 ± 2038
**24**	695	777	763	362	266	493	5704	1294 ± 1955
**25**	695	739	500	301	307	493	5994	1290 ± 2081
**1–25**	**1850**	**1600**	**1600**	**766**	**1118**	**1426**	**6498**	**2122 ± 1962**

Values are the mean daily running distance (meters/day) recorded for each animal in each week of voluntary wheel access; group values are mean ± SD (n = 7/group). The last row of each group (weeks 1-25) shows the mean daily distance of each animal over the whole 25-week intervention. No significant differences between PA-S and PA-IR were observed at any week (two-way ANOVA with Šídák's post-test; Figure 3C). Surgery (sham or ischemia-reperfusion) was performed only at the end of the intervention. PA-S (physically active-sham) and PA-IR (physically active-ischemia). SD, standard deviation.
